# Deciphering the Origin, Evolution, and Physiological Function of the Subtelomeric Aryl-Alcohol Dehydrogenase Gene Family in the Yeast Saccharomyces cerevisiae

**DOI:** 10.1128/AEM.01553-17

**Published:** 2017-12-15

**Authors:** Dong-Dong Yang, Gustavo M. de Billerbeck, Jin-jing Zhang, Frank Rosenzweig, Jean-Marie Francois

**Affiliations:** a LISBP, Université de Toulouse, CNRS, INRA, INSA, Toulouse, France; b School of Agriculture and Food Sciences, Zhejiang A & F University, Lin'an, China; c School of Biology, Georgia Institute of Technology, Atlanta, Georgia, USA; d INP-ENSAT, Castanet-Tolosan, France; e Toulouse White Biotechnology Center, Ramonville-Saint-Agne, France; University of Toronto

**Keywords:** aryl-alcohol dehydrogenases, AKR superfamily, subtelomeric, evolution, lignin, pseudogenization

## Abstract

Homology searches indicate that Saccharomyces cerevisiae strain BY4741 contains seven redundant genes that encode putative aryl-alcohol dehydrogenases (AAD). Yeast *AAD* genes are located in subtelomeric regions of different chromosomes, and their functional role(s) remain enigmatic. Here, we show that two of these genes, *AAD4* and *AAD14*, encode functional enzymes that reduce aliphatic and aryl-aldehydes concomitant with the oxidation of cofactor NADPH, and that Aad4p and Aad14p exhibit different substrate preference patterns. Other yeast *AAD* genes are undergoing pseudogenization. The 5′ sequence of *AAD15* has been deleted from the genome. Repair of an *AAD3* missense mutation at the catalytically essential Tyr^73^ residue did not result in a functional enzyme. However, ancestral-state reconstruction by fusing Aad6 with Aad16 and by N-terminal repair of Aad10 restores NADPH-dependent aryl-alcohol dehydrogenase activities. Phylogenetic analysis indicates that *AAD* genes are narrowly distributed in wood-saprophyte fungi and in yeast that occupy lignocellulosic niches. Because yeast *AAD* genes exhibit activity on veratraldehyde, cinnamaldehyde, and vanillin, they could serve to detoxify aryl-aldehydes released during lignin degradation. However, none of these compounds induce yeast *AAD* gene expression, and Aad activities do not relieve aryl-aldehyde growth inhibition. Our data suggest an ancestral role for *AAD* genes in lignin degradation that is degenerating as a result of yeast's domestication and use in brewing, baking, and other industrial applications.

**IMPORTANCE** Functional characterization of hypothetical genes remains one of the chief tasks of the postgenomic era. Although the first Saccharomyces cerevisiae genome sequence was published over 20 years ago, 22% of its estimated 6,603 open reading frames (ORFs) remain unverified. One outstanding example of this category of genes is the enigmatic seven-member *AAD* family. Here, we demonstrate that proteins encoded by two members of this family exhibit aliphatic and aryl-aldehyde reductase activity, and further that such activity can be recovered from pseudogenized *AAD* genes via ancestral-state reconstruction. The phylogeny of yeast *AAD* genes suggests that these proteins may have played an important ancestral role in detoxifying aromatic aldehydes in ligninolytic fungi. However, in yeast adapted to niches rich in sugars, *AAD* genes become subject to mutational erosion. Our findings shed new light on the selective pressures and molecular mechanisms by which genes undergo pseudogenization.

## INTRODUCTION

Functional characterization of predicted genes remains one of the chief tasks of the postgenomic era. Two decades after the initial release of the Saccharomyces cerevisiae genome (1), uncertainty remains as to the exact number of genes, the boundaries between them, and their function (2–7). According to the most recent estimates in the Saccharomyces Genome Database (SGD), the yeast genome consists of 6,603 open reading frames (ORFs), of which 4,848 are “verified,” 944 are “uncharacterized,” and 811 are “dubious.” Genes are considered verified once any of their products (transcript or protein) are detected, and indeed, many of these 4,848 genes have only been verified by global transcriptional analyses. To completely characterize the yeast genome and achieve an accurate assessment of gene content and function, high-throughput analyses must be complemented by careful case-by-case experimental analysis of those genes still classified as putative and those proteins still annotated as hypothetical. The yeast aryl-alcohol dehydrogenases (*AAD*) family is an outstanding example of this class of genes.

Prediction of the 7 putative aryl-alcohol dehydrogenases (*Aad*) in yeast was based on *in silico* analyses that showed ≥85% amino acid similarity to an aryl-alcohol dehydrogenase purified from the white rot fungus Phanerochaete chrysosporium (8–10). Aad proteins belong to the aldo-keto-reductase (AKR) superfamily, which has more than 190 members (11). AKR enzymes can reduce a variety of substrates, such as sugar aldehydes, keto-steroids, keto-prostaglandins, retinals, and quinones (11). AKR proteins are usually monomeric proteins of low molecular mass (in the range of 34 kDa) and have an (α/β)_8_-barrel motif, a conserved catalytic tetrad consisting of four amino acids (Tyr, Asp, Lys, and His), and a conserved cofactor binding domain for NAD(P)H (11). To explore the biological function of this gene family in S. cerevisiae, Delneri et al. constructed single, double, triple, quadruple, quintuple, sextuple, and septuple *aad* knockouts (12). Surprisingly, these mutants exhibited no pronounced phenotype when tested for their ability to mate, sporulate, or degrade aromatic aldehydes, and neither did they differ with respect to cell composition, in particular, ergosterol content and phospholipid profile (12). Later, the same authors reported that all putative *AAD* genes contained sequences similar to the oxidation-reactive yap1 transcriptional factor binding sites either upstream of or within their coding sequences; however, transcripts were only detectable for *AAD4* and *AAD6* under oxidation challenge by diamide, diethyl maleic acid ester, or H_2_O_2_ (13). While microarray analyses also suggest that putative Aad proteins may be involved in yeast's response to oxidative damage, heavy-metal stress, and certain fungicides (14–21), a mechanistic understanding of the role(s) they might play in doing so is lacking. To gain this understanding, detailed biochemical analyses are needed.

Previously, we showed that a homologue of yeast AAD, Phanerochaete chrysosporium Aad1p, can reduce aryl-aldehyde derivatives to their corresponding less-toxic alcohol forms (10). This result suggested that yeast Aad could serve to detoxify aromatic inhibitors produced in lignocellulosic ethanol production (22), as well as in the bioremediation of environmental pollutants, such as benzene, toluene, ethylbenzene and xylene (BTEX) derivatives (23). To explore these possibilities and to enlarge our understanding of the function and phylogeny of the yeast Aad gene family, we performed detailed biochemical and molecular genetic analyses. In this study, we show that only two genes of this family (*AAD4* and *AAD14*) encode enzymatic activities on aliphatic and aryl-aldehydes, whereas the other five putative members are being pseudogenized, a finding that informs our speculation about the evolutionary trajectory that created this subtelomeric gene family.

## RESULTS

### Among the seven-member yeast *AAD* gene family, only *AAD4* and *AAD14* encode functional aryl-aldehyde dehydrogenases.

**FIG 1:**
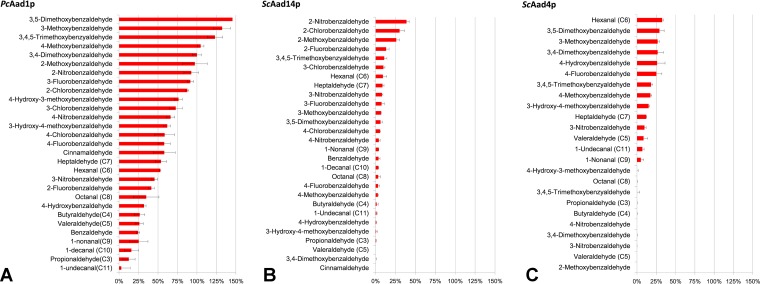
*Sc*Aad4p and *Sc*Aad14p have aryl-aldehyde reductase activity. Activities were assayed in morpholineethanesulfonic acid (MES) buffer (50 mM [pH 6.1]) containing 0.3 mM NADPH and 0.3 mM substrate. The standard activity of *Pc*Aad1p in reducing 3,4-dimethoxybenzaldehyde (being 5.4 μmol · min^−1^ · mg^−1^) was set at 100%. Data represent the means ± standard deviations of the results from triplicate experiments.

The NADPH-dependent P. chrysosporium Aad1 protein (*Pc*Aad1p) has an average of ∼85% residue similarity with all S. cerevisiae Aadp protein (*Sc*Aadp protein) members (sequence alignment shown in Fig. S1). We therefore adopted *Pc*Aad1p as a positive reference during the expression, purification, and enzymatic assay of the putative yeast *Sc*Aad proteins. Highly purified recombinant proteins were obtained following elution from glutathione affinity chromatography resin (Fig. S2). Enzymatic activity of the seven purified yeast Aad recombinant proteins showed that only *Sc*Aad14p and *Sc*Aad4p were able to reduce a group of candidate aryl-aldehydes with the consumption of NADPH (Fig. 1), validating their predicted enzyme category as aryl-alcohol dehydrogenases (EC 1.1.1.90). Under the same assay conditions, reference *Pc*Aad1p was active on a broader spectrum of aryl-aldehyde substrates than either *Sc*Aad14p or *Sc*Aad4p (Fig. 1). Notably, the reference *Pc*Aad1p and the active yeast *Sc*Aadp proteins shared substrate affinities for aliphatic and aryl-aldehydes with other previously reported aldo-keto reductase and aldehyde reductases (24–28) (Table 1). Interestingly, each of the two active *Sc*Aadp proteins demonstrated a unique pattern of substrate specificity. *Sc*Aad4p is more active on aryl-aldehyde-bearing substituents at positions 3, 4, and 5, or even those bearing double and triple substitutions on the aromatic ring, while *Sc*Aad4p was inactive on aryl-aldehydes that have position 2 substitutions (Fig. 1C). In contrast, *Sc*Aad14p exhibited its highest activity on aryl-aldehydes substituted at position 2 (Fig. 1B). Regarding catalytic efficiency, *Sc*Aad4p and *Sc*Aad14p have activity in the range of micromoles per minute per milligrams toward their preferred aryl-aldehyde substrates, e.g., 1.76 μmol · min^−1^ · mg^−1^ for *ScA*ad4p on hexanal and 2.10 μmol · min^−1^ · mg^−1^ for *Sc*Aad14p on 2-nitrobenzaldehyde. These values are 3-fold lower than the *Pc*Aad1p activity measured on 3,4-dimethoxybenzaldehyde.

**TABLE 1:** Yeast aldehyde reductases and their kinetic constants toward preferred substrates

Enzyme (reference)	Cofactor preference	*K_m_* (μM)	Aliphatic aldehyde substrate^*a*^			Aryl-aldehyde substrate^*a*^		
			Substrate	*K_m_* (μM)	*k*_cat_ (min^−1^)	Substrate	*K_m_* (μM)	*k*_cat_ (min^−1^)
*Pc*Aad1 (10)	NADPH	39	Hexanal	NA	247	3,4-Dimethoxybenzaldehyde	12	530
			Heptaldehyde	NA	138	Benzaldehyde	1,700	430
						Cinnamaldehyde	3,400	670
Aad4^*b*^	NADPH	NA	Hexanal	NA	172	3,4-Dimethoxybenzaldehyde	NA	142
			Heptaldehyde	NA	64	4-Nitrobenzaldehyde	NR	NR
Aad14^*b*^	NADPH	NA	Hexanal	NA	50	4-Nitrobenzaldehyde	NA	22
			Heptaldehyde	NA	46	Cinnamaldehyde	NR	NR
Aad10^-35C^^*b*^	NADPH	NA	Hexanal	NR	NR	4-Nitrobenzaldehyde	NR	NR
			Heptaldehyde	NR	NR	Cinnamaldehyde	NR	NR
Aad6^518G^^*b*^	NADPH	NA	Hexanal	NR	NR	4-Nitrobenzaldehyde	NR	NR
			Heptaldehyde	NR	NR	Cinnamaldehyde	NR	NR
Adh6 (25)	NADPH	29	Hexanal	152	21,270	Cinnamaldehyde	172	18,400
			Pentanal	60	22,700	Veratraldehyde	73	15,800
Adh7 (26)	NADPH	NA	Pentanal	49	11,915	Cinnamaldehyde	43	7,913
			3-Methylbutanal	48	9,581	Veratraldehyde	58	6,000
Gre3 (27)	NADPH	13	Hexanal	3,100	109	4-Nitrobenzaldehyde	120	142
Gcy1 (28)	NADPH	12	Butyraldehyde	5,400	81	4-Nitrobenzaldehyde	130	71
						Benzaldehyde	5,200	58
Ypr1 (29)	NADPH	8.7	Hexanal	390	354	4-Nitrobenzaldehyde	1,070	1,776
			2-Methylbutyraldehyde	1,090	524	9,10-Phenanthrequinone	2,600	272
Yjr096w (28)	NADPH	370	Butyraldehyde	1,800	0.5	4-Nitrobenzaldehyde	500	88
						Benzaldehyde	4,700	4
Ydl124w (28)	NADPH	23	Butyraldehyde	210,000	14	4-Nitrobenzaldehyde	30	3.3
						Benzaldehyde	240	4

a *k*_cat_ as reported or normalized based on reported *V*_max_. NA, data not available; NR, not reactive.

b Results of this study.

While *Pc*Aad1p can use both NADPH (*K_m_*, 39 μM) and NADH (*K_m_*, 220 μM), neither of the yeast Aad proteins was active with NADH as a reduction cofactor. *Pc*Aad1p can also act as an oxidase against several aliphatic and aromatic alcohols (at pH 10.3, with NADP^+^ as a cofactor). *Sc*Aad4p and *Sc*Aad14p showed no oxidation activities on any of the following alcohols: pentanol, hexanol, heptanol, 3,4-dimethoxybenzyl alcohol, benzyl alcohol, cinnamyl alcohol, 4-methoxybenzyl alcohol, 4-hydroxybenzyl alcohol, 3,5-dimethoxybenzyl alcohol, 3-hydroxy-4-methoxybenzyl alcohol, 4-hydroxy-3-methoxybenzyl alcohol (vanillyl alcohol), 3,4,5-trimethoxybenzyl alcohol, and 2-phenylethanol (2-tailed *t* test, *P* < 0.05; *n* = 3).

### In ancestral-state reconstruction, fusion of Aad6 and Aad16 results in an active Aad enzyme.

**FIG 2:**
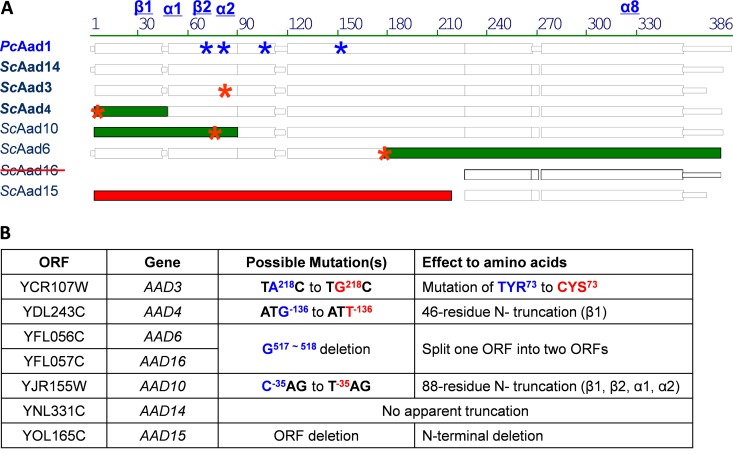
Single-base-pair substitutions, truncations, and deletions in the *AAD* gene family of yeast S288C. (A) Amino acid sequence alignments of *Pc*Aad1p (reference protein) and the seven putative *Sc*Aadp proteins. Blue asterisks (*) denote positions of four strictly conserved essential amino acids in *Pc*Aad1p: Asp^71^, Tyr^76^, Lys^103^, and His^152^. Structural details of the reference protein are provided in Fig. S4. Red asterisks (*) denote inferred point mutations to relative ancestral *ScAAD* genes. Green color denotes the truncated part of the ancestral gene. Point mutations result in (i) substitution of the conserved Tyr^73^ in *Sc*Aad3p, (ii) truncation of β-sheet 1 in *Sc*Aad4p, (iii) truncation of two α-helices and two β-sheets in *Sc*Aad10p, and (iv) the split of one ORF into two ORFs (*Sc*Aad6p and *Sc*Aad16p). The crossed-out *Sc*Aad16 indicates that this previously annotated ORF is a truncated part of AAD6 (in chromosome VI), not an independent ORF. The 5′ coding sequence of the *Sc*Aad15p was completely deleted from the genome of yeast lab strain S288C (in red). (B) Positions of the corresponding mutations at the nucleic acid and amino acid levels. Minus symbol (−) indicates nucleotide positions upstream of the ORFs as they were annotated in SGD at the time of submission.

Sequence alignment suggested that *AAD6* and *AAD16* were originally one open reading frame split into two by a nucleotide deletion in the *AAD6* coding sequence at positions G^517^ to C^518^ (Fig. 2 and S1). The insertion of a guanine nucleotide at this position places *AAD6* and *AAD16* in frame, which results in a new hypothetical protein (termed *Sc*Aad6^518G^p) that shares >80% amino acid sequence similarity with other Aad family members. We reconstructed the hypothetical ancestral state (29) of *Sc*Aad6^518G^p by site-directed mutagenesis and heterologously expressed the translation product as a glutathione *S*-transferase (GST) tag fusion protein in Escherichia coli, using the pGS-21a vector. The recombinant *Sc*Aad6^518G^p protein was purified to high homogeneity (Fig. S3) and subjected to enzymatic assay against the same panel of aryl-aldehydes described for *Pc*Aad1p (10). Assays of the purified protein yielded weak but detectable enzymatic activity toward several aldehydes, relative to the background. Kinetic (*K_m_* and *V*_max_) measurements on the reconstructed *Sc*Aad6^518G^p protein revealed affinities for phenylacetaldehyde (*K_m_* = 296 ± 30 μM) and 4-methoxybenzaldehyde (*K_m_* = 217 ± 26 μM). The maximum rate of reaction (*V*_max_) of *Sc*Aad6^518G^p on these substrates was one order of magnitude lower than that of *Pc*Aad1p (Table 2 and Fig. S5).

**TABLE 2:** Recovery of enzyme activity from pseudogenized *Sc*Aad10p and *Sc*Aad6/16p following ancestral state reconstruction^*a*^

Enzyme	*Pc*Aad1p		*Sc*Aad10^-35C^p		*Sc*Aad6^518G^p	
	*V*_max_	*K_m_*	*V*_max_	*K_m_*	*V*_max_	*K_m_*
4-Methoxybenzaldehyde	6.63 ± 0.12	87.3 ± 116	0.08 ± 0.01	157 ± 39.8	0.55 ± 0.02	217 ± 26.1
Hydroxymethylfurfural	2.40 ± 0.27	171 ± 36.7	0.37 ± 0.07	608 ± 246	NR	
Phenylacetaldehyde	9.00 ± 0.27	527 ± 72.5	0.30 ± 0.08	17.6 ± 2.20	0.49 ± 0.02	296 ± 29.6

a *V*_max_ (in micromoles per minute per milligram) and *K_m_* (micromolar) values are shown as the mean ± standard error (SE) of the results from triplicate experiments. NR, not reactive.

### Tyr^76^ is essential for the function of *Pc*Aad1p but is missing in *Sc*Aad3p.

We identified the catalytic tetrad (Asp^71^, Tyr^76^, Lys^103^, and His^152^) in the reference enzyme *Pc*Aad1p (Fig. S4). However, alignment showed that in *Sc*Aad3p, a cysteine^73^ residue was present at the corresponding catalytic site tyrosine^73^. This substitution in *Sc*Aad3p could have been caused by a single nucleotide mutation from A^218^ to G^218^ of its coding sequence (Fig. 2 and S1). We therefore performed two site-directed mutagenesis experiments. In *Pc*Aad1p, the functional Tyr^76^ was mutated into Cys^76^ (TG^227^C to TA^227^C), whereas in *Sc*Aad3p, the Cys^73^ was replaced by Tyr^73^ (TA^218^C to TG^218^C). Following heterologous expression and purification, the activities of the recombinant proteins were assayed on a variety of aryl-aldehyde substrates. As expected, the *Pc*Aad1^Tyr76Cys^p variant was completely inactive on all of these substrates; however, correction of the missense mutation in *Sc*Aad^Cys73Tyr^p failed to produce a functional enzyme (data not shown).

### In ancestral-state reconstruction, repair of the *Sc*Aad10p N-terminal domain results in an enzyme having aryl-aldehyde activity.

We failed to detect any catalytic activity for the putative *Sc*Aad10p. However, sequence alignment suggested that a 264-bp sequence upstream of the SGD-annotated *ScAAD10* may have once been in frame (detailed alignment is shown in Fig. S1), interrupted by a T^−35^AG-to-C^−35^AG substitution, counting from the SGD-annotated start codon. This point mutation (denoted by asterisk in Fig. 2) may have introduced a nonsense mutation in the ancestral *ScAAD10*. According to our structural modeling of *Pc*Aad1p (see Fig. S4), the truncated 88 residues contain the β1, β2, α1, and α2 domains of the classical (α_8_β_8_) motif, as well as two essential amino acids (Asp^71^ and Tyr^76^) found in all aldo-keto reductases. We therefore investigated whether replacing T by C at position −35 from ATG could resurrect an active Aad10 protein. The reconstructed polypeptide, *Sc*Aad10^−35T^p, was expressed in E. coli as a GST-tagged protein and purified to near homogeneity (see Fig. S3), and its kinetics were tested on a variety of aryl-aldehydes. The reductase activity of the reconstructed *Sc*Aad10^−35C^ protein was observed on hydroxymethylfurfural, phenylacetaldehyde, and 4-methoxybenzaldehyde with NADPH as a cofactor. In all instances, the *V*_max_ values of reconstructed *Sc*Aadp were (10 to 80 times) lower than those of *Pc*Aad1p, and in 4 of 5 instances, the *K_m_* values for these substrates were higher than that of the *Pc*Aad1p (Table 2). Interestingly, reconstructed *Sc*Aad10p exhibits an even higher affinity for phenylacetaldehyde (*K_m_*, 17.6 μM) than does *Pc*Aad1p, suggesting that perhaps phenylacetaldehyde was a native substrate for *Sc*Aad10p at some point in its evolutionary past.

### Yeast *AAD* gene expression is not induced by aryl-aldehydes, and *AAD* overexpression does not increase yeast resistance to these compounds.

Aromatic aldehyde derivatives are abundant in nature, being released as saprophytes degrade and digest plant matter (23, 30, 31). These aldehydes also occur in industrial decomposition of lignocellulosic biomass, where they inhibit yeast growth (32–35). We confirmed this inhibitory effect for four aryl-aldehydes: veratraldehyde, hydroxymethylfurfural, vanillin, and *trans*-cinnamaldehyde, at concentrations ranging from 25 to 50 mM (data not shown). As *Sc*Aadp proteins have the capacity to transform these compounds into less-toxic alcoholic derivatives, we investigated whether exposure of cells to aryl-aldehydes could induce *AAD* gene expression. Contrary to expectation, none of the seven *AAD* transcript levels increased following 1 to 2 h treatment with aryl-aldehydes, relative to a no-aldehyde control (2-tailed *t* test, *P* < 0.05; *n* = 6).

To test whether overexpression of *AAD* genes conferred higher resistance of yeast to aryl-aldehyde toxicity, we individually subcloned *ScAAD3*, *ScAAD4*, *ScAAD14*, and *PcAAD1* into the multicopy YEplac195 plasmid, placing each under the control of the strong constitutive PGK1 promoter. These constructs were then transformed into wild-type BY4741 as well as into an *adh6* knockout, as the expression of Adh6 has been shown by itself to confer aryl-aldehyde resistance (36). None of the transformants exhibited growth improvement on the four aryl-aldehydes, even those bearing *AAD1* from P. chrysosporium. Indeed, while activity in BY4741 cell crude extracts expressing *Pc*Aad1p was 2-fold higher than that of the blank-plasmid control, activities in cells expressing *Sc*Aad3p, *Sc*Aad4p, and *Sc*Ad14p did not significantly differ (Fig. S7). We did not test whether the overexpression of reconstructed Aad proteins improved growth on aryl-aldehyde, as the *in vitro* activity of resurrected Aad proteins is even lower than those of *Sc*Aad4p and *Sc*Aad14p.

### Across sequenced S. cerevisiae genomes, *AAD* genes vary in both copy number and nucleotide sequence.

Our observation that the majority of BY4741 *AAD* genes were undergoing pseudogenization prompted us to survey their distribution and sequence variation in diverse sequenced strains, including those adapted to the laboratory (BY4741 and Sigma1278b), to wine fermentation (EC1118, AWRI1631, AWRI796, Lalvin QA23, and VL3), to the brewing of beer (Fosters B and Fosters O) and sake (Kyokai no. 7), and to life as an opportunistic human pathogen (YJM789) (Table 3). Using reconstructed full-length *AAD* genes as query sequences, our analysis uncovered extensive variation in *AAD* copy number as well as in the number and location of *AAD* single nucleotide polymorphisms (SNPs). These polymorphisms may be related to the ecology of the species in which they are found. For example, compared to lab strain BY4741, industrial yeasts have one to five *AAD* homologs missing from their genomes: in Fosters B and Fosters O, which are used for beer production, 5 of 7 *AAD* genes are missing, while in the wine yeast Lalvin QA23, *AAD6*, *AAD16*, and *AAD15* are missing. Unlike the missense or nonsense mutations in *ScAAD3*, *ScAAD10*, and *ScAAD6/16*, the 5′ coding sequence of *AAD15* has been deleted from the BY4741 genome. *AAD15* was entirely absent from the genomes of eight of 11 yeast conspecifics. BY4741 *AAD6/AAD16* was not observed in any other genome, although a full-length *AAD6* was detected in S. cerevisiae strain T7, which was isolated from oak tree exudate (Fig. S6).

**TABLE 3:** *AAD* ORFs are highly varied among sequenced S. cerevisiae genomes

Strain	Nucleic acid sequence similarity/amino acid sequence similarity (%)^*a*^										
	Lab		Pathogen	Wine					Beer		Sake
	BY4741	Sigma1278b	YJM789	EC1118	AWRI1631	AWRI796	Lalvin QA23	VL3	Fosters B	Fosters O	Kyokai no. 7
*AAD3*	100/100	98.2/82.7	96.4/93.7	99.8/99.5	99.9/99.5	99.4/82.1	99.7/67.6	99.8/85.2	NR	NR	NR
*AAD4^-136G^*	99.9/83.5	NR	93.2/93.9	94.3/84.6	94.4/84.3	94.3/84.3	94.2/71.3	94.2/84.3	95.9/84.3	94.3/84.3	93.4/94.7
*AAD6^518G^*	99.9/-	NR	NR	NR	NR	NR	NR	NR	NR	NR	99.8/-
*AAD10^-35C^*	99.9/76.4	87.3/55.2	99.9/76.4	99.7/76.4	99.8/76.1	99.6/99.5	NR	92.6/72.9	NR	NR	*/*
*AAD14*	100/100	100/100	99.2/99.5	97.1/97.6	NR	97.1/97.6	96.9/52.0	96.9/65.3	97.3/97.1	97.5/97.3	100/100
*AAD15*	100/100	100/100	NR	97.7/95.1	NR	NR	NR	NR	NR	NR	NR

a Nucleic acid/amino acid sequence similarities are relative to query sequences from lab strain S288C. NR, *AAD* homologs were not retrieved. *, percentage similarity not calculated when only partial sequences were found at the end of a sequencing contig with missing 3' or 5' coding sequences. (Note: high similarity in nucleic acids can result in low amino acid similarity due to ORF truncation.)

### *AAD* gene phylogeny indicates preferential distribution among wood-saprophyte fungi.

**FIG 3:**
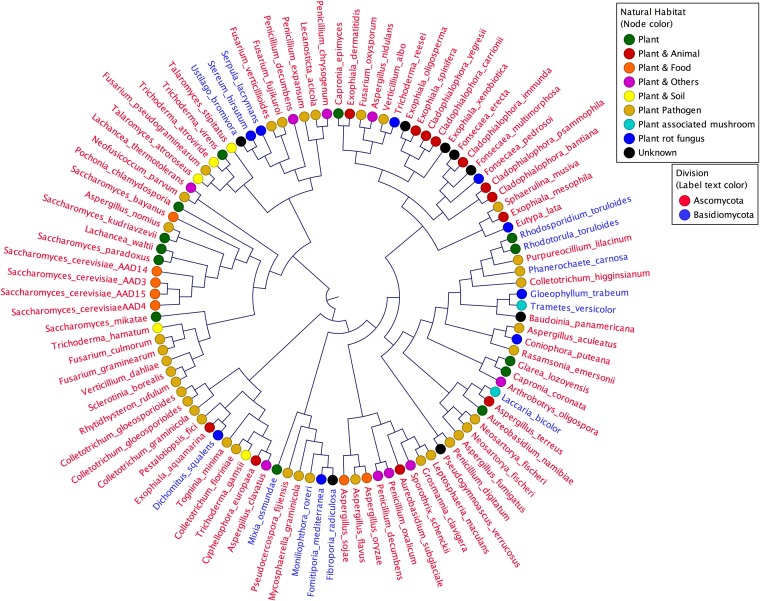
*AAD* orthologs are distributed within Basidiomycota and Ascomycota fungi typically associated with plant habitats. Orthologs were identified by BLAST using yeast *AAD14* as the query sequence against the NCBI database. Phylogeny of the resulting sequences was constructed using a *k*-mer (*k* = 15)-based neighbor-joining method.

**FIG 4:**
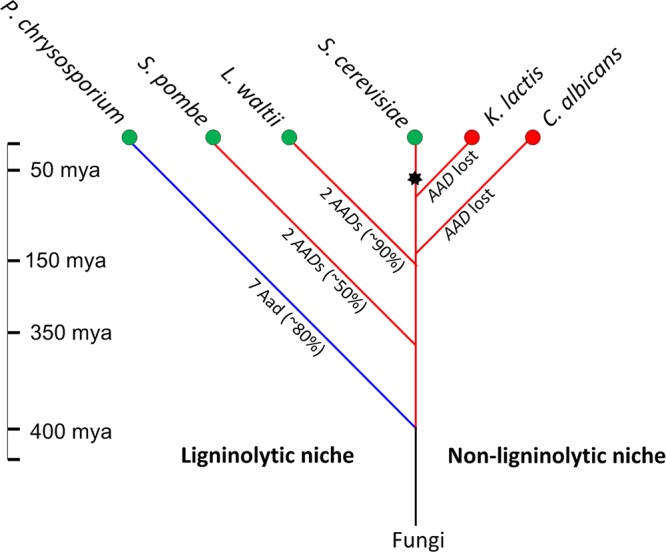
Hypothesized evolution of fungal aryl-aldehyde reductases, enzymes that detoxify lignin by-products. *AAD* orthologs are observed among wood and leaf litter saprophytes but not among fungi adapted to animal niches. Four hundred million years ago, split of Basidiomycota (blue branch) and the Ascomycota (red branches) (37) coincides with emergence of woody plants in the form of gymnosperms (56–58); 350 mya, divergence of S. pombe and Saccharomyces spp. (42); 150 mya, divergence of L. waltii and S. cerevisiae (44); 50 to 100 mya, adaptation to sugar-rich niches via *Adh* neofunctionalization (black asterisk) and ethanologenesis (22, 68, 69, 70). Green nodes denote species found in ligninolytic habitats and red nodes denote species found in nonligninolytic habitats. Text along the branches signifies *AAD* copy numbers and their average nucleotide similarity to *ScAAD14*.

*ScAAD14* encodes a full-length active protein whose sequence is highly similar (>80%) to other predicted yeast *AAD* genes. We therefore conducted a BLASTn search (expected [Exp.] threshold = 10; score, ≥60) for *ScAAD14* orthologs in the NCBI database. An unrooted phylogeny (Fig. 3) shows that the most closely related 101 *AAD* orthologs fall into the Ascomycota and the Basidiomycota, groups that shared a common ancestor 400 million years ago (mya) (37). Species harboring *AAD* orthologs include lignin-scavenger specialists, such as the white rot fungus Phanerochaete carnosa, the poroid crust fungus Dichomitus squalens, and the mushroom Trametes versicolor, as well as plant pathogens, such as Neofusicoccum parvum (38–41). About one-third of species harboring *AAD* orthologs are yeast or filamentous fungi found in plant-associated habitats, such as wood debris, leaves, or bark exudate. Two *AAD* orthologs were retrieved from the millet-isolated fission yeast Schizosaccharomyces pombe, which diverged from the Saccharomyces lineage around 350 mya (Fig. 4) (42). Orthologs exhibiting 90% amino acid sequence similarity to *Sc*Aad14p were retrieved from the Mochi tree-isolated (43) pre-whole-genome duplication (WGD) yeast Lachancea waltii, which diverged from the S. cerevisiae progenitor 150 mya (44). Multiple *AAD* homologs are distributed among the Saccharomyces sensu stricto species S. bayanus, S. kudriavzevii, S. mikatae, and S. paradoxus. Significantly, all *AAD*-bearing species were isolated from environments rich in decaying plant matter, such as leaves and oak bark (45–49). *AAD* genes are altogether missing from yeast that have either colonized animal hosts (e.g., Candida glabrata) or have been domesticated for dairy production (Kluyveromyces lactis).

## DISCUSSION

Based on their homology to *AAD1* in the white-rot fungus P. chrysosporium, seven *AAD* genes are predicted to occur in the genome of S. cerevisiae strain BY4741 (8, 12). However, to date, the function(s) of these genes have proven elusive. Here, we show that two members of this family, *ScAAD4* and *ScAAD14*, encode functional enzymes that reduce aryl-aldehydes to their corresponding aryl-alcohols with NADPH as a cofactor. *Sc*Aad4p and *Sc*Aad14p exhibit overlapping but nonidentical substrate preferences, as often seen when genes arise via duplication from a common ancestor (50, 51). Other members of the BY4741 *AAD* gene family appear to be undergoing pseudogenization, and the mechanisms by which this is likely occurring offer clues as to how protein function might be restored. *In silico* analysis of extant *AAD* coding and protein sequences, coupled with three-dimensional (3D) modeling of enzymatically active *Pc*Aad1p suggested that a split ORF had inactivated *AAD6/AAD16*, that an N-terminal deletion had inactivated *AAD10*, and that a missense mutation at a key catalytic residue had abolished *AAD3*-encoded activity. Ancestral-state reconstruction (29) via site-directed mutagenesis enabled us to resurrect aryl-aldehyde dehydrogenase activity for two of these *Sc*Aad proteins (*Sc*Aad6^518G^p and *Sc*Aad10^−35C^p).

Pseudogenization of *AAD* genes is also evident in the extensive polymorphism observed among other strains of S. cerevisiae isolated from diverse habitats. Gene copy number and gene sequence are more variable in subtelomeric *AAD* genes relative to nonsubtelomeric aldehyde reductases, such as *ADH* genes and *AKR* genes listed in Table S1. We suggest that pseudogenization of *AAD* genes is facilitated by their location, as genes located in subtelomeric regions undergo both meiotic and mitotic recombination at elevated rates relative to genes located elsewhere in the genome (52). Indeed, subtelomeric instability has been hypothesized as a mechanism that accelerates adaptation via rapid functional divergence of novel alleles (53). Thus, in yeast, the present distribution of highly polymorphic and variably functional *AAD* genes reflects their evolutionary history.

S. cerevisiae Aad *in vitro* activities are about 3-fold lower than those of *Pc*Aad1p from the saprophytic white rot fungus Phanerochaete chrysosporium. Also, they are one to two orders of magnitude lower than the broad-specificity NADPH-dependent aldehyde reductases encoded by S. cerevisiae aldehyde dehydrogenase VI (*ADH6*, ca. 183 μmol · min^−1^ · mg^−1^ for cinnamaldehyde) (24) and aldehyde dehydrogenase VII (*ADH7*, ca. 90 μmol · min^−1^ · mg^−1^ for cinnamaldehyde) (25). *ScAAD* expression is not induced by aryl-aldehydes, and *ScAAD* overexpression does not confer resistance to toxic aryl-aldehyde derivatives. The data presented in Table 1 suggest that the capacity of *Sc*Aadp proteins to detoxify aldehydes has been superseded by promiscuous yeast aldehyde reductases encoded by members of the Akr and Adh families. Unlike yeast *AAD* genes, yeast *ADH* genes are transcriptionally upregulated in the presence of such compounds (36), and overexpression of *ADH6* and *ADH7* increases yeast resistance to veratraldehyde, anisaldehyde, and 5-hydroxymethylfurfural (24, 36). Yeast aldehyde reductases catalyze diverse reactions that lead to ethanol production as well as to the reduction of branched-chain and aromatic aldehydes into fusel alcohols via the Ehrlich pathway (54). Concerning the biological function of yeast Aadp proteins, we note a lack of consensus as to whether they play a role in the production of aroma metabolites arising from the Ehrlich pathway. Dickinson et al. (55) found that none of the *AAD* genes could be implicated in fusel alcohol formation from amino acids, while Styger et al. attributed to *AAD6* the aromatic profile peculiar to fermentation in the presence of excess branched amino acids: leucine, valine, and isoleucine (56). As the yeast strain used by Styger et al. (56) was isogenic to BY4741 but opposite in its mating type, their finding is inconsistent with our data, which indicate that *AAD6* does not encode a functional enzyme.

The fact that few yeast Aad enzymes can reduce toxic aromatic aldehydes released by lignin depolymerization (23, 30, 31), coupled with the findings that yeast *AAD* genes are undergoing pseudogenization and that *AAD* orthologs are restricted to fungi in ligninolytic niches, leads us to hypothesize that Aad function originated among wood saprophytes. Consistent with this hypothesis, our phylogenetic analyses indicate that the orthologs most closely related to S. cerevisiae *AAD* are confined to the Basidiomycota and Ascomycota (Fig. 3). These phyla diverged about 400 mya (37), contemporaneous with the earliest fossil record of wood (56–58), suggesting that ancestral *AAD* genes arose about the same time that lignin became abundant in the terrestrial environment. The hemiascomyte clade that encompasses the saccharolytic yeasts (22, 59) emerged ∼150 mya, broadly coincidental with the rise of fruit-forming angiosperms (22, 60–63) (Fig. 4). We therefore propose that extant yeast Aad proteins are largely evolutionary relics, albeit ones that bear witness to fungal ancestors that invaded lignocellulosic niches created in the mid-Devonian with the rise of vascular plants on dry land.

### Conclusion.

The role played by the subtelomeric *AAD* gene family in the yeast Saccharomyces cerevisiae has been obscure, as there is no obvious mutant phenotype and no documented enzymatic activity. Here, we report that of seven members of this family present in lab strain BY4741, only *AAD4* and *AAD14* encode proteins able to reduce aryl-aldehydes to their corresponding alcohols using NADPH as a cofactor. Among the five remaining putative *AAD* genes, aryl-aldehyde dehydrogenase activity could be resurrected in Aad10p after its N terminus was restored and in Aad6/Aad16p after two coding regions were fused. Aryl-aldehyde dehydrogenase activity could not be resurrected from either *AAD3* or *AAD15*. Phylogenetic data suggest that *AAD* genes originated in wood-saprophytic fungi. The likely ancestral function of Aad proteins was to reductively detoxify aromatic aldehydes arising from lignin depolymerization. In contrast, the yeast ancestors of Saccharomyces cerevisiae adapted to sugar-rich niches that arose following the advent of fruit-bearing angiosperms. Ethanologenic fermentation favored the evolution of multiple alcohol dehydrogenases via duplication and neofunctionalization. Yeast *ADH* genes also encode aldehyde reductase activities, and these have played increasingly important roles in redox balance and detoxification, compensating for the loss of Aad activity through pseudogenization.

## MATERIALS AND METHODS

### Strains and media.

Unless otherwise stated, S. cerevisiae BY4741 (*MAT***a** *his3*Δ*1 leu2*Δ*0 met15*Δ*0 ura3*Δ*0*) was used as the host strain in all cloning, overexpression, and biochemical studies. The S. cerevisiae *adh6* mutant (*MAT***a** *his3Δ1 leu2*Δ*0 met15*Δ*0 ura3*Δ*0* Δ*adh6*, BY4741 background) from the Yeast Knockout collection was included in detoxification assays. Yeast were cryopreserved as −80°C glycerol stocks and routinely propagated in either liquid or solid YPD medium (1% yeast extract, 2% peptone, 2% glucose) at 30°C. Experimental assays were performed using synthetic uracil drop-out (URA^−^) medium [1.7 g/liter yeast nitrogen base without amino acids, 5 g/liter (NH_4_)_2_SO_4_, 20 g/liter glucose, 1.92 g of uracil drop-out supplements (catalog no. Y1501; Sigma-Aldrich)] containing 76 mg/liter uracil (catalog no. U1128; Sigma-Aldrich) if required for growth. E. coli strain BL21 Star (catalog no. C6020-03; Invitrogen) was used to host heterologous expression and purification of recombinant Aad proteins. Luria broth and plate medium (10 g/liter tryptone, 5 g/liter yeast extract, 10 g/liter NaCl) was used for routine propagation of E. coli strains with 150 μg · ml^−1^ ampicillin, 50 μg · ml^−1^ kanamycin, or 34 μg · ml^−1^ chloramphenicol added for plasmid selection.

### Bioinformatics. (i) Identification of ORF truncation and deletion.

Open reading frames plus 1-kb up- and downstream the sequences of the seven yeast *AAD* genes were retrieved from the Saccharomyces Genome Database (SGD), as well the *PcAAD1* coding sequence; these were imported to VectorNTI Advanced 10.0 software (Invitrogen) for alignment. The inference of ORF truncation for *ScAAD4*, *ScAAD10*, *ScAAD6*, and *ScAAD16* and the ORF deletion of *ScAAD15* was based on the fact that (i) the out-of-frame nucleic acid sequences share ≥80% consensus positions (Fig. S1), (ii) a simulated single-base-pair mutation put all of the hypothetical coding sequences back in frame, (iii) in-frame sequences are similar in length to the coding sequence of the reference gene *PcAAD1*, and (iv) all hypothetical S. cerevisiae ORFs (*Sc*ORFs) encode comparably sized proteins that share ≥80% amino acid sequence similarity (Fig. S1).

### (ii) Search for AAD homologs.

The SGD-retrieved ORFs of *ScAAD3* and *ScAAD14* and hypothetical ORFs of *ScAAD4*, *ScAAD10*, *ScAAD6/16*, and *ScAAD15* were used as query sequences to perform a standard WU-BLASTN search against the genome data of Saccharomyces cerevisiae species (https://www.yeastgenome.org/blast-fungal), using default parameters. The retrieved subject sequences and their corresponding scaffold sequences were exported to VectorNTI Advanced 10.0 for further validation. *AAD* homologues were accepted as valid only if the chromosome numbers initially annotated by the sequencing project and the alignment between the exported scaffold and reference S288C sequences did not suggest any chromosome rearrangement. Nucleic acid and translated sequences of *AAD* homologues from 10 representative S. cerevisiae species were aligned with that of the reference S288C strain. Sequence alignments, oligonucleotide thermal properties, protein molecular weight (MW) calculation, and design of plasmids and primers were conducted using the software VectorNTI Advanced 10.0 (Invitrogen).

### (iii) Search for *AAD* orthologs.

The *ScAAD14* coding sequence was used as the query sequence to perform a BLASTn against nucleotide collection (nr/nt) genome databases (max target = 5,000, Exp. threshold = 10) at https://blast.ncbi.nlm.nih.gov/Blast.cgi. The matched *AAD* ortholog sequences were ranked by scores. For multiple orthologs from one species, only the first sequence (score, ≥60; length, >150 bp) was chosen to represent the corresponding species. A *k*-mer (*k* = 15)-based neighbor-joining tree construction was performed in the CLC Genomics Workbench 10 software.

### (iv) Modeling of *Pc*Aad1p structure.

*In silico* modeling of *Pc*Aad1p structure was performed using the YASARA Structure software (YASARA Biosciences) with the resolved AKR11C1 structure as the template (64). Assessment of modeling quality was carried out using the SWISS-MODEL Web server (http://swissmodel.expasy.org/). The *Pc*Aad1p sequence with key amino acids and motifs thereby revealed was aligned with yeast Aadp proteins (Fig. 2).

### Cloning, heterologous expression, and purification of recombinant yeast Aadp proteins.

PCR-based amplification of S. cerevisiae *AAD* genes was carried out on purified genomic BY4741 DNA using the forward and reverse primers described in Table 4. Fifty-microliter PCRs were performed using the Phusion PCR system (catalog no. F630S; Thermo Fisher; containing 100 ng of DNA, 0.2 mM dinucleoside triphosphate [dNTP], 1.5 mM MgCl_2_, 0.5 μM reverse and forward primers, 1 unit of neuraminidase [NA] polymerase), as follows: 1 cycle of 98°C for 30 s, 30 cycles of 98°C for 10 s, 65°C for 30 s, and 72°C for 45 s; and 1 cycle of 72°C for 7 min. The primers described in Table 4 generate PCR amplicons flanked by KpnI and NotI restriction sites at their 5′ and 3′ ends, respectively. Amplicons were A-tailed and ligated into pGEM-T Easy vectors (catalog no. A1360; Promega). Following transformation into E. coli DH5α and growth under ampicillin selection, positive clones were verified by double enzyme digestion and Sanger sequencing. KpnI-NotI fragments were then excised from the T-vectors and religated into plasmid pGS-21a (catalog no. SD0121; GenScript) previously linearized by KpnI-NotI digestion. Successful constructs were verified by plasmid sequencing and then transformed into E. coli BL21 Star as pGS-21a-*Sc*Aad3, pGS-21a-*Sc*Aad4, and pGS-21a-*Sc*Aad14. Expression and purification of recombinant yeast Aad proteins were carried out in parallel with reference protein *Pc*Aad1p, using procedures previously described for *Pc*Aad1p (10). Assays on reconstructed yeast *Sc*Aad3^Cys73Tyr^p, Aad6/16^518G^p, and Aad10^−35C^p proteins were carried out in parallel with a purified His_6_-GST tag.

**TABLE 4:** Primers and plasmids for cloning, plasmid construction, and site-directed mutagenesis

Primer	Primer sequence^*a*^	Purpose(s)	Yielded plasmid(s)
AAD3_pGS_F	GGTACC*GACGACGACGACAAG***ATG**ATTGGGTCCGCGTCCG	*ScAAD3* cloning to pGS-21a	pGS-21a-*Sc*Aad3
AAD3_pGS_R	GCGGCCGCAACATTATTCGTACCATATTT		
AAD4_pGS_F	GGTACC*GACGACGACGACAAG***ATG**GGCTCTATGAATAAGGAACA	*ScAAD4* cloning to pGS-21a	pGS-21a-*Sc*Aad4
AAD4_pGS_R	GCGGCCGCATCGAAGGAAATCTGCGCA		
AAD14_pGS_F	GGTACC*GACGACGACGACAAG***ATG**ACTGACTTGTTTAAACCTCT	*ScAAD14* cloning to pGS-21a	pGS-21a-*Sc*Aad14
AAD14_pGS_R	GCGGCCGCATTGTCAAAAGCTATCCTGGCA		
PC_AAD_ORF1_F1_HR	GTAATTATCTACTTTTTACAACAAATATAAAACAAGATCTCGACTCTAGAGGATCC**ATG**AACATCTGGGCACCCG	Subcloning *PcAAD1* from pGS-21a-*Pc*Aad1 (Yang et al. [10]), for purpose of overexpression *PcAAD1* in yeast BY4747	YEplac195PGK/CYC1-JL52-URA3-*Pc*Aad1
PC_AAD_ORF1_R1_HR	CCAAAGGCCATCTTGGTACCGGGCCCCCCCTCGAGGTCGACGGTATCGATAAGCTT**CTA**CTTCTGGGGGCGGATAGC		
SC_AAD3_F1_HR	GTAATTATCTACTTTTTACAACAAATATAAAACAAGATCTCGACTCTAGAGGATCC**ATG**ATTGGGTCCGCGTCCG	Subcloning *ScAAD3* from pGS-21a-S*c*Aad3, for purpose of overexpression *ScAAD3* in yeast BY4747	YEplac195PGK/CYC1-JL52-URA3-*Sc*Aad3
SC_AAD3_R1_HR	CCAAAGGCCATCTTGGTACCGGGCCCCCCCTCGAGGTCGACGGTATCGATGCGGCCGC**CTA**AACATTATTCGTACCATATTT		
SC_AAD4_F1_HR	GTAATTATCTACTTTTTACAACAAATATAAAACAAGATCTCGACTCTAGAGGATCC**ATG**GGCTCTATGAATAAGGAACA	Subcloning *ScAAD4* from pGS-21a-S*c*Aad4, for purpose of overexpression *ScAAD4* in yeast BY4747	YEplac195PGK/CYC1-JL52-URA3-*Sc*Aad4
SC_AAD4_R1_HR	CCAAAGGCCATCTTGGTACCGGGCCCCCCCTCGAGGTCGACGGTATCGATGCGGCCGC**TTA**ATCGAAGGAAATCTGCGCA		
SC_AAD14_F1_HR	GTAATTATCTACTTTTTACAACAAATATAAAACAAGATCTCGACTCTAGAGGATCC**ATG**ACTGACTTGTTTAAACCTCT	Subcloning *ScAAD14* from pGS-21a-S*c*Aad14, for purpose of overexpression *ScAAD14* in yeast BY4747	YEplac195PGK/CYC1-JL52-URA3-*Sc*Aad14
SC_AAD14_R1_HR	CCAAAGGCCATCTTGGTACCGGGCCCCCCCTCGAGGTCGACGGTATCGATGCGGCCGC**CTA**ATTGTCAAAAGCTATCCTGGCA		
PcAad1Tyr76mut2Cys_F1	AACTTCATTGATACCGCTAATGTCT**G**CCAAGACGAGACATCCGAGGAATTT	*Pc*Aad1p tyrosine^73^→cysteine^73^ mutagenesis	pGS-21a-*Pc*Aad1^Tyr76Cys^
PcAad1Tyr76mut2Cys_R1	AAATTCCTCGGATGTCTCGTCTTGG**C**AGACATTAGCGGTATCAATGAAGTT		
ScAad3MutCys2Tyr_BamHI_A1	ATCGCGCGGATCC**ATG**ATTGGGTCCGCGTCCGACTCATCTAGC	*Sc*Aad3p cysteine^73^→tyrosine^73^ mutagenesis	pGS-21a-*Sc*Aad1^Cys73 Tyr^
ScAad3MutCys2Tyr_B1	CCATTCTTCTGATTGCTCGTTTTGG**T**AGTTGTTTGCGGCATCAATGAAATT		
ScAad3MutCys2Tyr_C1	AATTTCATTGATGCCGCAAACAACT**A**CCAAAACGAGCAATCAGAAGAATGG		
ScAad3MutCys2Tyr_XhoI_D1	ATCGCCGCTCGAGAACATTATTCGTACCATATTTTTGAGTCAAGG		
ScAad6InserG_F1_A	ATCGATCGCGCGGATCC**ATG**GCTGATTTATTTGCTCCTGCTCC	Fusion of *ScAAD6-AAD16*	pGS-21a-ScAad6^518G^
ScAad6InserG_R1_B	AGACACACCCAAATAGAGGACCTTG**C**CCTGCTGCACTAGAATGTGTAAACT		
ScAad6InserG_F2_C	AGTTTACACATTCTAGTGCAGCAGG**G**CAAGGTCCTCTATTTGGGTGTGTCT		
ScAad6InserG_R2_D	ATCGATCGCCGCTCGAG**TTA**ATCGAAGGAAATCTGCGCAGACATTGC		
ScAad10Mute_F1_A	ATCGATCGCGCGGATCC**ATG**TCTGAGGCTTTTGGACCTGCAC	*Sc*Aad10^-35C^p N truncation repair	pGS-21a-*Sc*Aad10^C-35^
ScAad10Mute_R1_B	ATCCAAGTCTCTGACTGCTCATACTGATAATTATTTGCAGTATCAATGAAATTTCC		
ScAad10Mute_F2_C	GGAAATTTCATTGATACTGCAAATAATTATCAGTATGAGCAGTCAGAGACTTGGAT		
ScAad10Mute_R2_D	ATCGATCGCCGCTCGAG**CTA**ATCTTCGAAGCTAATCTTGGCA		

a Italics indicate the enterokinase-coding sequence, boldface indicates start and stop codons, and underlining indicates restriction sites.

### Subcloning of *AAD* into the YEplac195PGK/CYC1-JL52-URA3 yeast vector.

The coding sequence of *Pc*Aad1p was PCR amplified from plasmid pGS-21a-*Pc*Aad1 using primer set PC_AAD_ORF1_F1_HR and PC_AAD_ORF1_R1_HR in a 50-μl Phusion PCR (see Table 4 for primer sequences). This primer set generates amplicons flanked by 50-bp sequences homologous to the YEplac195PGK/CYC1-JL52-URA3 vector at multiple cloning sites. The purified PCR amplicon and BamHI- and HindIII-linearized YEplac195PGK/CYC1-JL52-URA3 were cotransformed to S. cerevisiae strain BY4741 using the standard lithium acetate protocol (65). Yeast transformants obtained on synthetic URA^−^ plates were subjected to plasmid isolation and sequencing. The resulting plasmid was named YEplac195PGK/CYC1-JL52-URA3-PaAad1 (see Table 4). Yeast *AAD* genes were cloned into plasmid YEplac195PGK/CYC1-JL52-URA3 via homologous recombination using methods similar to those described above for *PcAAD1*. The primers used are described in Table 4.

### Site-directed mutagenesis and reconstruction of hypothetical Aad ORFs. (i) Reconstruction of truncated *Sc*Aad6p and *Sc*Aad16p.

Bioinformatic analyses indicated that extant yeast genes *AAD6* and *AAD16* together once formed a functional open reading frame. To reconstruct this presumed ancestral *AAD6/AAD16*, we used a three-step PCR-based procedure. The first reaction uses S. cerevisiae BY4741 genomic DNA as the template with primers *Sc*Aad6InserG_F1_A and *Sc*Aad6InserG_R1_B, of which *Sc*Aad6InserG_R1_B carries a G insert at positions G^517∼518^. The second reaction uses the same template but with primer set ScAad6InserG_F2_C and ScAad6InserG_R2_D, which produces an amplicon that contains the G insert and also overlaps with the first amplicon. The two amplicons were then fused together by an overlapping PCR that uses primers ScAad6InserG_F1_A and ScAad6InserG_R2_D. The details for overlapping PCR and subsequent ligation are similar to those described above for *Sc*Aad^C−35^p. The resulting construct was verified by sequencing and termed pGS-21a-ScAad6^518G^p.

### (ii) Mutation of PcAad1^Tyr76Cys^p.

PCR-based site-directed mutagenesis (SDM) was performed on previously described plasmid pGS-21a-PcAad1 (10) to generate an A→G mutation that would replace the presumed catalytic tyrosine^76^ (coded by TAC) with a cysteine^76^ (coded by TGC). The *Pc*Aad1Tyr76mut2Cys_F1 and *Pc*Aad1Tyr76mut2Cys_R1 primers (0.5 μM working concentration each, both having the A→G mutation, as listed in Table 4) and 20 ng of plasmid were used in a 50-μl Phusion PCR. The PCR conditions consisted of one cycle of denaturation at 98°C for 60 s, followed by 20 cycles of amplification (98°C denaturation for 15 s, 50°C annealing for 30 s, and 72°C extension for 4 min), and one final extension at 72°C for 7 min. Two microliters of DpnI and 5 μl of 1× CutSmart buffer (catalog no. R0176S; New England BioLabs) were added to the completed reaction and incubated at 37°C for 1 h, after which an additional 2 μl of DpnI was added to completely digest the methylated template plasmid. The resulting reaction mixture was purified and then transformed to E. coli strain DH5α. Transformants were subjected to plasmid purification and sequencing to verify the presence of the mutation. The newly constructed plasmid was named pGS-21a-*Pc*Aad1^Tyr76Cys^.

### (iii) Mutation of ScAad3p.

PCR-based site-direct mutagenesis was performed to mutate cystine^73^ (coded by TG^218^C) into a presumed functional tyrosine^73^ (coded by TAC) in a manner similar to that described above for *Pc*Aad1^Cys76^p, except that primer set *Sc*Aad3MutCys2Tyr_B1/*Sc*Aad3MutCys2Tyr_C1 was used against template vector pGS21a-*Sc*Aad3. The resulting constructs were screened by colony PCR using the primer set AAD3_pGS_F/AAD3_pGS_R, validated by Sanger sequencing, and then named pGS21a-*Sc*Aad3^Cys73Tyr^.

### (iv) Repairing N-terminal truncation of *Sc*Aad10p.

Reconstruction of the presumed *Sc*Aad10p ancestor by repair of its N-terminal truncation was carried out using three rounds of PCR. The first reaction used S. cerevisiae genomic DNA as the template in conjunction with primers *Sc*Aad10Mute_F1_A and *Sc*Aad10Mute_R1_B, of which *Sc*Aad10Mute_R1_B carries a mutated C^−35^AG codon that replaces the presumed premature Stop codon T^−35^AG. The second reaction uses the same genomic template, but primer set ScAad10Mute_F2_C and ScAad10Mute_R2_D, which produces an amplicon with the corrected C^−35^AG codon and overlaps with the first amplicon. The third reaction is an overlapping PCR that uses purified amplicons from the first two rounds as templates in conjunction with primer set *Sc*Aad10Mute_F1_A and *Sc*Aad10Mute_R2_D. The first two reactions use standard the Phusion protocol described above but with annealing at 65°C. The overlapping Phusion PCR cycling conditions were 1 cycle at 95°C for 4 min, followed by 25 cycles of 95°C for 30 s, 68°C for 30 s, and 72°C for 3 min. The resulting amplicon was precipitated, digested with BamHI and Xhol, and then gel purified and ligated with similarly linearized pGS-21a. The new construct was verified by Sanger sequencing and termed pGS-21a-*Sc*Aad10^C−35^.

### Analysis of *AAD* expression in response to aromatic aldehyde exposure.

Three independent colonies of S. cerevisiae strain BY4741 were picked from YPD agar, inoculated in 5 ml of YPD broth, and cultured overnight at 30°C, with shaking at 150 rpm. Half a milliliter of these overnight cultures was used to inoculate 180 ml of YPD broth in 1-liter Erlenmeyer flasks. Yeast cultures were grown in triplicate at 30°C at 150 rpm, and their optical density (OD) was recorded at λ = 600 nm. Aliquots of stock solutions of 3,4-dimethoxybenzaldehyde, hydroxymethylfurfural, 4-hydroxy-3-methoxybenzaldehyde, and *trans*-cinnamaldehyde were added to mid-log yeast cultures (OD, 0.8) to yield final concentrations of 25 mM, 25 mM, 25 mM, and 50 mM, respectively. Untreated cultures were included as a negative control. Cells were harvested 1 h and 2 h following treatment by centrifuging 45 ml of culture at 10,000 × *g* and 4°C for 5 min. The resulting pellets were immediately frozen in liquid nitrogen and stored at −80°C.

Yeast mRNA was extracted following the yeast RNA extraction protocol provided in the SV Total RNA isolation system kit (catalog no. Z3100; Promega). RNA quality was assessed via Bioanalyzer 2100 using the RNA 6000 Nano LabChip kit (Agilent Technologies, Massy, France) and quantified in a NanoDrop ND-1000 UV-visible light spectrophotometer (Fisher Scientific SAS, Illkirch, France). cDNA was synthesized from 1 μg of total RNA in 20-μl reaction mixtures using the iScript cDNA synthesis kit (catalog no. 1708891; Bio-Rad).

Due to high sequence homology among *AAD* family members, two sets of gene-specific primers (GSP) were designed for each *AAD* gene (Table 5**)**. Housekeeping genes *TAF10*, *TFC1*, and *UBC6* were used as internal controls for the normalization of expression data (66). The binding efficiencies of the primers were evaluated using the series dilution method (67). Real-time PCRs were carried out using a MyiQ single-color real-time PCR detection system (catalog no. 170-9740; Bio-Rad, France). Reactions were set up in triplicate for each of three biological replicates to ensure the reliability of the results. The reactions were performed in a 25-μl final reaction volume using iQ SYBR green Supermix (Bio-Rad), under previously described reaction conditions (10).

**TABLE 5:**
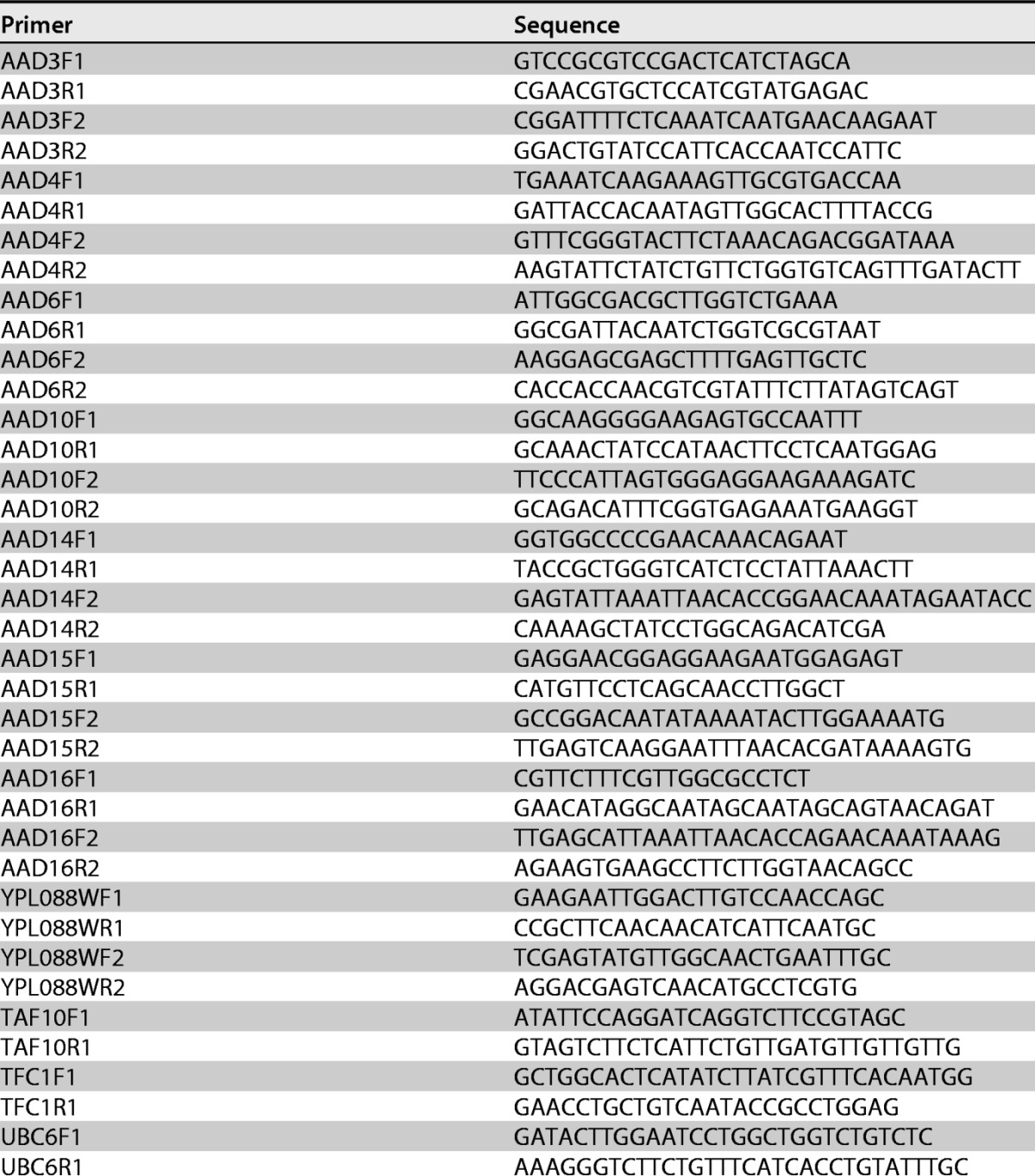
List of primers for quantitative PCR for expression studies

### Plate assay of aldehyde-mediated growth inhibition.

Multicopy yeast expression vectors YEplac195PGK/CYC1-JL52-URA3 (harboring either *ScAAD3*, *ScAAD4*, *ScAAD14*, or *PcAAD1*) were transformed into S. cerevisiae strain BY4741 and the Yeast Knockout (YKO) *adh6* (clone no. 6460, *MAT***a** *his3*Δ*1 leu2*Δ*0 met15*Δ*0 ura3*Δ*0* Δ*adh6*) mutant using the standard lithium-acetate procedure (65). Three independent transformants were picked and used to inoculate replicate overnight cultures. The next day, 10 μl from these cultures was used to inoculate 5 ml of URA^−^ medium, which was grown at 30°C and 150 rpm. At an OD at 600 nm (OD_600_) of 1, the culture was 10^5^-fold diluted in URA^−^ medium at 25°C. Two microliters of diluted cell suspensions was pipetted onto URA^−^ agar containing one of the four aldehydes (3,4-dimethoxybenzaldehyde, hydroxymethylfurfural, 4-hydroxy-3-methoxybenzaldehyde, or *trans*-cinnamaldehyde) at concentrations of 0, 6.25, 12.5, or 50 mM. Colonies appearing on these plates were photographed every 12 h over the course of 72 h.

### Analysis of AAD enzyme substrate specificity and enzymatic kinetics.

Heterologous expression, purification, and biochemical characterization of yeast Aad proteins were carried out in parallel, with the *Pc*Aad1p serving as a reference. *Pc*Aad1p and all recombinant yeast Aad proteins bear an N-terminal His_6_-GST and a C-terminal His_6_ tag and were purified following the GST-affinity batch purification (10). The activities of purified reference *Pc*Aad1p, yeast Aadp proteins, and mutated yeast Aad recombinant proteins were assayed against a panel of aliphatic/aromatic aldehydes and aryl-alcohols using both NAD(P)H and NAD(P)^+^ as reduction and oxidation cofactors (10). Reactions were quantified spectrophotometrically by following the consumption or production of cofactor NAD(P)^+^(H) at λ = 340 nm (ε_340_ = 6.2 mM^−1^ · cm^−1^) in 250-μl microplates containing 0.3 mM cofactor and 0.3 mM substrates (10) (microplate reader model 680XR; Bio-Rad). Kinetic parameters (*K_m_* and *V*_max_) quantifying NADP^+^(H) consumption/formation were assayed at 355 nm (ε_355_ = 5.12 mM^−1^ · cm^−1^) using a UV-visible spectrophotometer (Shimadzu UV1800) in 1-ml cuvettes, as described previously; the substrate absorption at this wavelength is negligible (10). For each recombinant protein, significant differences relative to a pGS-21a blank plasmid were calculated using a 2-tailed *t* test at a *P* value of <0.05.

## Supplementary Material

Supplemental material
