# Supplementary material for: Deciphering the Origin, Evolution, and Physiological Function of the Subtelomeric Aryl-Alcohol Dehydrogenase Gene Family in the Yeast Saccharomyces cerevisiae
Source: Appl Environ Microbiol. 2017 Dec 15;84(1):e01553-17. doi: 10.1128/AEM.01553-17 (PMC5734042; doi:10.1128/AEM.01553-17)
Supplement: Supplemental material [file supp_84_1_e01553-17__index.html]

Deciphering the Origin, Evolution, and Physiological Function of the Subtelomeric Aryl-Alcohol Dehydrogenase Gene Family in the Yeast Saccharomyces cerevisiae — Supplemental material 

# Deciphering the Origin, Evolution, and Physiological Function of the Subtelomeric Aryl-Alcohol Dehydrogenase Gene Family in the Yeast Saccharomyces cerevisiae

## Supplemental material

- Supplemental file 1 -

  Nucleotide and amino acid sequence alignments (Fig. S1); heterologous expression and GST-affinity batch purifications (Fig. S2 and S3); structural analysis (Fig. S4); recovery of enzyme activity (Fig. S5); full-length *AAD6* identified in T7 living in oak tree exudate (Fig. S6); aldehyde reduction activity (Fig. S7); variability of *AAD* ORFs (Table S1).

  PDF, 891K
