# Supplementary material for: Deciphering the Origin, Evolution, and Physiological Function of the Subtelomeric Aryl-Alcohol Dehydrogenase Gene Family in the Yeast Saccharomyces cerevisiae
Source: Appl Environ Microbiol. 2017 Dec 15;84(1):e01553-17. doi: 10.1128/AEM.01553-17 (PMC5734042; doi:10.1128/AEM.01553-17)
Supplement: Supplemental material [file AEM.01553-17_zam001188238s1.pdf]

FIG S1 (A)

```
ScAAD10       (1) ----------------------------------------------------------------------------------------------------
ScAAD10-+1kb  (695) GAATAGC-TTCATAACCCACCGCCGCAAAA-TGTGCTCTTCGAAATGTCTGAGG-CTTTTGGACCTGCACCTGAACCACCTACCGAGTTAGGACGTCTTA
ScAAD15       (1) ----------------------------------------------------------------------------------------------------
ScAAD15-+1kb  (332) TTGAGAATGAGATTTTGACACCATTACTAATAATCCTATTCACTAATACTTACTGTAAGCGGTA----GAAAAACACATGTA-CAAGAAA----------
ScAAD3-+1kb   (951) TTCA--ATTAC-TCTTTCGATCATCTAGAAGAAGCATATCCA-TCGTGCTTAAAATGATTGGGTCCGCGTCCGACTCATCTAGCAAGTTAGGACGCCTCC
ScAAD3        (1) ------------------------------------------------------ATGATTGGGTCCGCGTCCGACTCATCTAGCAAGTTAGGACGCCTCC
ScAAD16       (1) ----------------------------------------------------------------------------------------------------
ScAAD16-+1kb  (291) CTGAATA-TCTCTTCTATATCATTTTCAAT-AGCGATTTTTAATATGGCTGATT-TATTTGCTCCTGCTCCTGAACCATCTACAGAGTTAGGACGTCTCA
ScAAD6-+1kb   (959) CTGAATA-TCTCTTCTATATCATTTTCAAT-AGCGATTTTTAATATGGCTGATT-TATTTGCTCCTGCTCCTGAACCATCTACAGAGTTAGGACGTCTCA
ScAAD6        (1) --------------------------------------------ATGGCTGATT-TATTTGCTCCTGCTCCTGAACCATCTACAGAGTTAGGACGTCTCA
ScAAD14       (1) --------------------------------------------ATGACTGACT-TGTTTAAACCTCTACCTGAACCACCTACCGAATTGGACGTCTCA
ScAAD14-+1lb  (959) TACTTAA-CATATTGTCTATTGCATAAAAA-ACCACTATTCAGCATGACTGACT-TGTTTAAACCTCTACCTGAACCACCTACCGAATTGGACGTCTCA
ScAAD4        (1) ----------------------------------------------------------------------------------------------------
ScAAD4-+144kb (819) CACTAGAATACACTTCCTATAGCTACAAAATATTATTCAATAATATTAGTGAAT-TATTTAAACCTCTACCTGAACCACCTACCGAGTTGGACGTCTCA

ScAAD10       (1) ----------------------------------------------------------------------------------------------------
ScAAD10-+1kb  (792) GGGTCCTATCTAAAACAGCTGGTATAAGAGTTTCTCCACTAATCCTGGGAGGTATGTCTATTGGTGACGCCTGGTCTGGATTCATGGGATCAATGGACAA
ScAAD15       (1) ----------------------------------------------------------------------------------------------------
ScAAD15-+1kb  (417) --TTGGCTGCTGAAA-TGCTGCCATGGATTCAACCACAGTGAAAAAGGTTAAGTGGAA-ACGT--GATAAACTGCGGCCTGCTTGCTCGCGATGC-CAA
ScAAD3-+1kb  (1047) GATTTCTTTCTGAAACTGCCGCTATTAAAGTATCCCCGTTAATCCTAGGAGAAGTCTCATACGATGGAGCACGTTCGGATTTTCTCAAATCAATGAACAA
ScAAD3       (47) GATTTCTTTCTGAAACTGCCGCTATTAAAGTATCCCCGTTAATCCTAGGAGAAGTCTCATACGATGGAGCACGTTCGGATTTTCTCAAATCAATGAACAA
ScAAD16       (1) ----------------------------------------------------------------------------------------------------
ScAAD16-+1kb  (388) GAGTTCTTTCTAAAAGTGCTGGTATTAGAGTTTCCCCTCTCATTTTGGGAGGAATGTCAATTGGCGACGCTTGGTCTGAAATCCTAGGATCAATGAGCAA
ScAAD6-+1kb  (1056) GAGTTCTTTCTAAAAGTGCTGGTATTAGAGTTTCCCCTCTCATTTTGGGAGGAATGTCAATTGGCGACGCTTGGTCTGAAATCCTAGGATCAATGAGCAA
ScAAD6       (56) GAGTTCTTTCTAAAAGTGCTGGTATTAGAGTTTCCCCTCTCATTTTGGGAGGAATGTCAATTGGCGACGCTTGGTCTGAAATCCTAGGATCAATGAGCAA
ScAAD14      (56) GGGTTCTTTCTAAAACTGCCGGCATAAGGGTTTCACCGCTAATTCTGGGAGGAGCTTCAATCGGCGACGCATGGTCAGGCTTTATGGGCTCTATGAATAA
ScAAD14-+1lb (1056) GGGTTCTTTCTAAAACTGCCGGCATAAGGGTTTCACCGCTAATTCTGGGAGGAGCTTCAATCGGCGACGCATGGTCAGGCTTTATGGGCTCTATGAATAA
ScAAD4        (1) -----------------------------------------------------------------------------------ATGGGCTCTATGAATAA
ScAAD4-+1kb   (918) GGGTTCTTTCTAAAACTGCCGGCATAAGAGTTTCACCGCTAATTCTGGGAGGAGCTTCAATCGGCGATGCATGGTCAGGCTTTATGGGCTCTATGAATAA

ScAAD10       (1) ----------------------------------------------------------------------------------------------------
ScAAD10-+1kb  (892) AGAACAAGCTTTTGAGCTACTTGATGCTTTTTACCAAGCAGGCGGAAATTTCATTGATACTGCAAATAATTATTTA---GTATGA--GCAGTCAGAGACTT
ScAAD15       (1) ----------------------------------------------------------------------------------------------------
ScAAD15-+1kb  (510) CAAAATTCATTACG----GTGTGAATATGAAGAGAGAACAGATTTATCTGCCAATGTTGCAGCAAACGACTCTGATGGATTCAATTCCTCTCATAAGC-T
ScAAD3-+1kb  (1147) GAATCGAGCTTTTGAATTGCTTGATACTTTTTACGAGGCAGGTGGAAATTTCATTGATGCCGCAAACAACTGCCA---AAACGA--GCAATCAGAAGAAT
ScAAD3       (147) GAATCGAGCTTTTGAATTGCTTGATACTTTTTACGAGGCAGGTGGAAATTTCATTGATGCCGCAAACAACTGCCA---AAACGA--GCAATCAGAAGAAT
ScAAD16       (1) ----------------------------------------------------------------------------------------------------
ScAAD16-+1kb  (488) GGAGCGAGCTTTTGAGTTGCTCGATGCTTTTTATGAGGCAGGTGGAAATTTCATTGATACTGCAAATAATTATCA---AAACGA--ACAGTCAGAAGCTT
```

```
ScAAD6-+1kb  (1156) GGAGCGAGCTTTTGAGTTGCTCGATGCTTTTTATGAGGCAGGTGGAAATTTCATTGATACTGCAAATAATTATCA---AAACGA--ACAGTCAGAAGCTT
     ScAAD6   (156) GGAGCGAGCTTTTGAGTTGCTCGATGCTTTTTATGAGGCAGGTGGAAATTTCATTGATACTGCAAATAATTATCA---AAACGA--ACAGTCAGAAGCTT
    ScAAD14   (156) GGAACAGGCCTTTGAACTTCTTGATGCTTTTTATGAAGCTGGAGGTAATTGTATTGATACTGCAAACAGTTACCA---AAATGA--AGAGTCAGAGATTT
ScAAD14-+1lb (1156) GGAACAGGCCTTTGAACTTCTTGATGCTTTTTATGAAGCTGGAGGTAATTGTATTGATACTGCAAACAGTTACCA---AAATGA--AGAGTCAGAGATTT
      ScAAD4   (18) GGAACAGGCTTTTGAACTTCTTGATGCTTTTTATGAAGCAGGAGGTAATTGCATTGATACTGCAAACAGTTACCA---AAATGA--AGAGTCAGAGATTT
ScAAD4-+1kb  (1018) GGAACAGGCTTTTGAACTTCTTGATGCTTTTTATGAAGCAGGAGGTAATTGCATTGATACTGCAAACAGTTACCA---AAATGA--AGAGTCAGAGATTT

     ScAAD10    (1) --------------ATGGCATCAA-GAAAACTGCGTGACCAGATTGTAATTGCCACTAAATTTACCACGGATTATAAGGGTATGATGTAGGCAAGG-GG
ScAAD10-+1kb  (987) GGATCGGTGAATGGATGGCATCAA-GAAAACTGCGTGACCAGATTGTAATTGCCACTAAATTTACCACGGATTATAAGGGTATGATGTAGGCAAGG-GG
     ScAAD15    (1) ----------------------------------------------------------------------------------------------------
ScAAD15-+1kb  (605) CAATTTCGAACAGCAACCTGTACTTGAAAGGACTGGGCTTAGATATTCCTTACAAGT--GCCTGAAGGTGTCGTTAA----TACTACGCTGTCGATATGG
ScAAD3-+1kb  (1242) GGATTGGTGAATGGATACAGTCCA-GAAGGTTACGTGATCAAATTGTCATTGCAACCAAGTTTATAAAAAGCGATAAAAAGTATAAAGCAGGTGAAA-GT
      ScAAD3  (242) GGATTGGTGAATGGATACAGTCCA-GAAGGTTACGTGATCAAATTGTCATTGCAACCAAGTTTATAAAAAGCGATAAAAAGTATAAAGCAGGTGAAA-GT
     ScAAD16    (1) ----------------------------------------------------------------------------------------------------
ScAAD16-+1kb  (583) GGATCGGTGAATGGATGGTTTCAA-GAAAATTACGCGACCAGATTGTAATCGCCACCAAGTTTACCACTGACTATAAGAAATACGACGTTGGTGGTG-GT
ScAAD6-+1kb  (1251) GGATCGGTGAATGGATGGTTTCAA-GAAAATTACGCGACCAGATTGTAATCGCCACCAAGTTTACCACTGACTATAAGAAATACGACGTTGGTGGTG-GT
      ScAAD6  (251) GGATCGGTGAATGGATGGTTTCAA-GAAAATTACGCGACCAGATTGTAATCGCCACCAAGTTTACCACTGACTATAAGAAATACGACGTTGGTGGTG-GT
     ScAAD14  (251) GGATAGGTGAATGGATGGCATCAA-GAAAACTGCGTGACCAGATTGTAATTGCCACCAAGTTTACCGGAGATTATAAGAAGTATGAAGTAGGTGGTG-GT
ScAAD14-+1lb (1251) GGATAGGTGAATGGATGGCATCAA-GAAAACTGCGTGACCAGATTGTAATTGCCACCAAGTTTACCGGAGATTATAAGAAGTATGAAGTAGGTGGTG-GT
      ScAAD4  (113) GGATAGGTGAATGGATGAAATCAA-GAAAGTTGCGTGACCAAATTGTAATTGCCACCAAGTTTACCGGAGATTATAAGAAGTATGAAGTAGGTGGCG-GT
ScAAD4-+1kb  (1113) GGATAGGTGAATGGATGAAATCAA-GAAAGTTGCGTGACCAAATTGTAATTGCCACCAAGTTTACCGGAGATTATAAGAAGTATGAAGTAGGTGGCG-GT

     ScAAD10   (85) AAGAGTGCCAATTTCTGTGGGAATCACAAGCGCAGTTTGCATGTAAGTGTGAGAGATTCCCTTCGTAAGTTG-----CAAACTG---ATTGGATTGATAT
ScAAD10-+1kb (1085) AAGAGTGCCAATTTCTGTGGGAATCACAAGCGCAGTTTGCATGTAAGTGTGAGAGATTCCCTTCGTAAGTTG-----CAAACTG---ATTGGATTGATAT
     ScAAD15    (1) ----------------------------------------------------------------------------------------------------
ScAAD15-+1kb  (699) AACGCCGAAGA-TATGCTAGTTATAGTAGGATTAGTC-ACATTTCTGGATTATCCTTTTGCTGCGCATAGTCTGGCGCAACATGACCACTATATCAGGGC
ScAAD3-+1kb  (1340) AACACTGCCAACTACTGTGGTAATCACAAGCGTAGTTTACATGTGAGTGTGAGGGATTCTCTCCGCAAA-TT--GC--AAACTG---ATTGGATTGATAT
      ScAAD3  (340) AACACTGCCAACTACTGTGGTAATCACAAGCGTAGTTTACATGTGAGTGTGAGGGATTCTCTCCGCAAA-TT--GC--AAACTG---ATTGGATTGATAT
     ScAAD16    (1) ----------------------------------------------------------------------------------------------------
ScAAD16-+1kb  (681) AAAAGCGCAAACTACTGTGGCAATCACAAGCGTAGTTTGCATGTGAGTGTGAGGGATTCTCTCCGCAAATTG-----CAAACTG---ATTGGATTGATAT
ScAAD6-+1kb  (1349) AAAAGCGCAAACTACTGTGGCAATCACAAGCGTAGTTTGCATGTGAGTGTGAGGGATTCTCTCCGCAAATTG-----CAAACTG---ATTGGATTGATAT
      ScAAD6  (349) AAAAGCGCAAACTACTGTGGCAATCACAAGCGTAGTTTGCATGTGAGTGTGAGGGATTCTCTCCGCAAATTG-----CAAACTG---ATTGGATTGATAT
     ScAAD14  (349) AAAAGTGCCAACTACTGTGGTAATCACAAGCGTAGTTTACATGTGAGTGTGAGGGATTCTCTCCGCAAATTG-----CAAACTG---ATTGGATTGATAT
ScAAD14-+1lb (1349) AAAAGTGCCAACTACTGTGGTAATCACAAGCGTAGTTTACATGTGAGTGTGAGGGATTCTCTCCGCAAATTG-----CAAACTG---ATTGGATTGATAT
      ScAAD4  (211) AAAAGTGCCAACTATTGTGGTAATCACAAGCATAGTTTACATGTGAGTGTGAGGGATTCTCTCCGCAAATTG-----CAAACTG---ATTGGATTGATAT
ScAAD4-+1kb  (1211) AAAAGTGCCAACTATTGTGGTAATCACAAGCATAGTTTACATGTGAGTGTGAGGGATTCTCTCCGCAAATTG-----CAAACTG---ATTGGATTGATAT

     ScAAD10  (177) TCTTTACGTTCACTGGT--GGGATTATATGAGCTCCATTGAGGAAGTTATGGATAGTTTGCACATTCTTGTGCAG-CAGGGCAAAGTACTCTATCTAGGT
ScAAD10-+1kb (1177) TCTTTACGTTCACTGGT--GGGATTATATGAGCTCCATTGAGGAAGTTATGGATAGTTTGCACATTCTTGTGCAG-CAGGGCAAAGTACTCTATCTAGGT
     ScAAD15    (1) ----------------------------------------------------------------------------------------------------
ScAAD15-+1kb  (797) ACTTTGTGCTTCGTTGTACGGCATGGCGCTTGTTGACTTTAGCAATT-ATGCTAA--TGGTAT-TCCTTGTGAAGACACATCAAGAAG-TATACTAGGAC
```

```
ScAAD3-+1kb (1432) ACTTTACGTTCACTGGT--GGGATTATATGAGTTCAATCGAAGAATTTATGGATAGTTTGCATATTCTGGTCCAG-CAGGGCAAGGTCCTCTATTTGGGT
      ScAAD3 (432) ACTTTACGTTCACTGGT--GGGATTATATGAGTTCAATCGAAGAATTTATGGATAGTTTGCATATTCTGGTCCAG-CAGGGCAAGGTCCTCTATTTGGGT
       ScAAD16 (1) ----------------------------------------------------------------------------------------------------
ScAAD16-+1kb (773) ACTTTACGTTCACTGGT--GGGATTATATGAGTTCTATCGAAGAAGTTATGGATAGTTTACACATTCTAGTGCAG-CAGG-CAAGGTCCTCTATTTGGGT
ScAAD6-+1kb (1441) ACTTTACGTTCACTGGT--GGGATTATATGAGTTCTATCGAAGAAGTTATGGATAGTTTACACATTCTAGTGCAG-CAGG-CAAGGTCCTCTATTTGGGT
      ScAAD6 (441) ACTTTACGTTCACTGGT--GGGATTATATGAGTTCTATCGAAGAAGTTATGGATAGTTTACACATTCTAGTGCAG-CAGG-CAAGGTCCTCTATTTGGGT
     ScAAD14 (441) ACTTTACATTCACTGGT--GGGATTATATGAGTTCAATCGAAGAAGTTATGGATAGTTTGCATATTTTAGTTCAG-CAGGGCAAGGTCCTATATTTAGGA
ScAAD14-+1lb (1441) ACTTTACATTCACTGGT--GGGATTATATGAGTTCAATCGAAGAAGTTATGGATAGTTTGCATATTTTAGTTCAG-CAGGGCAAGGTCCTATATTTAGGA
      ScAAD4 (303) ACTTTACGTTCACTGGT--GGGATTATATGAGTTCAATCGAAGAAGTTATGGATAGTTTGCATATTTTAGTTCAG-CAGGGCAAAGTCCTCTATTTGGGT
ScAAD4-+1kb (1303) ACTTTACGTTCACTGGT--GGGATTATATGAGTTCAATCGAAGAAGTTATGGATAGTTTGCATATTTTAGTTCAG-CAGGGCAAAGTCCTCTATTTGGGT

     ScAAD10 (274) GTGTCTGATACTCCTGCCTGGGTTGTTTCTGCAGCAAATTACTACGCCACATCTCATGGTAAAACTCCCTTTAGTATCTATCAAGGTAAATGGAATGTAT
ScAAD10-+1kb (1274) GTGTCTGATACTCCTGCCTGGGTTGTTTCTGCAGCAAATTACTACGCCACATCTCATGGTAAAACTCCCTTTAGTATCTATCAAGGTAAATGGAATGTAT
       ScAAD15 (1) ----------------------------------------------------------------------------------------------------
ScAAD15-+1kb (892) CATTGTCATTCATAGAAAAGGCCATTTTTAGACGGATAGAAC-------------ATAGTAAG---CAATTTCGAGTTCAGCC-------TGGAACGTGT
ScAAD3-+1kb (1529) GTATCTGATACACCTGCTTGGGTTGTTTCTGCGGCAAACTACTACGCTACATCTTATGGTAAAACTCCCTTTAGTATCTACCAAGGTAAATGGAACGTGT
      ScAAD3 (529) GTATCTGATACACCTGCTTGGGTTGTTTCTGCGGCAAACTACTACGCTACATCTTATGGTAAAACTCCCTTTAGTATCTACCAAGGTAAATGGAACGTGT
       ScAAD16 (1) ----------------------------------------------------------------------------------------------------
ScAAD16-+1kb (869) GTGTCTGATACGCCTGCCTGGGTTGTTTCTGCGGCAAATTACTACGCTAAATCTCATGGTAAAACCCCTTTTAGCATCTATCAAGGTAAATGGAACCTGT
ScAAD6-+1kb (1537) GTGTCTGATACGCCTGCCTGGGTTGTTTCTGCGGCAAATTACTACGCTAAATCTCATGGTAAAACCCCTTTTAGCATCTATCAAGGTAAATGGAACCTGT
      ScAAD6 (537) GTGTCTGATACGCCTGCCTGGGTTGTTTCTGCGGCAAATTACTACGCTAAATCTCATGGTAAAACCCCTTTTAGCATCTATCAAGGTAAATGGAACCTGT
     ScAAD14 (538) GTATCTGATACACCTGCTTGGGTTGTTTCTGCGGCAAATTACTACGCTACATCTCATGGTAAAACTCCTTTTAGCGTCTATCAAGGTAAATGGAATGTAT
ScAAD14-+1lb (1538) GTATCTGATACACCTGCTTGGGTTGTTTCTGCGGCAAATTACTACGCTACATCTCATGGTAAAACTCCTTTTAGCGTCTATCAAGGTAAATGGAATGTAT
      ScAAD4 (400) GTGTCTGATACACCTGCTTGGGTTGTTTCTGCGGCAAACTACTACGCCACATCTCATGGGAAAACTCCTTTTAGTATCTATCAAGGTAAATGGAATGTGT
ScAAD4-+1kb (1400) GTGTCTGATACACCTGCTTGGGTTGTTTCTGCGGCAAACTACTACGCCACATCTCATGGGAAAACTCCTTTTAGTATCTATCAAGGTAAATGGAATGTGT

     ScAAD10 (374) TGAACAGGGACTTTGAACGTGATATCATTCCAATGGCTAGGCATTTTGGTATGGCTCTTGCTCCATGGGATGTTATGGAGGCGGGAGATTTCAGAGTAA
ScAAD10-+1kb (1374) TGAACAGGGACTTTGAACGTGATATCATTCCAATGGCTAGGCATTTTGGTATGGCTCTTGCTCCATGGGATGTTATGGAGGCGGGAGATTTCAGAGTAA
       ScAAD15 (1) --------------------------------ATGGCTAGGCATTTCGGTATGGCCCTCGCCCCATGGGATGTCATGGAGGTGGAAGATTTCAGAGTAA
ScAAD15-+1kb (969) TGAACAGAGATTTTGAGCGTGATATTATTCCAATGGCTAGGCATTTCGGTATGGCCCTCGCCCCATGGGATGTCATGGAGGTGGAAGATTTCAGAGTAA
ScAAD3-+1kb (1629) TGAACAGAGATTTTGAGCGTGATATTATTCCAATGGCTAGGCATTTCGGTATGGCCCTCGCCCCATGGGATGTCATGGAGGTGGAAGATTTCAGAGTAA
      ScAAD3 (629) TGAACAGAGATTTTGAGCGTGATATTATTCCAATGGCTAGGCATTTCGGTATGGCCCTCGCCCCATGGGATGTCATGGAGGTGGAAGATTTCAGAGTAA
       ScAAD16 (1) --------------------------------ATGGCTAGGCATTTCGGTATGGCCCTCGCCCCATGGGATGTCATGGAGGTGGAAGATTTCAGAGTAA
ScAAD16-+1kb (969) TGAACAGAGACTTCGAACGTGATATCATTCCAATGGCTAGGCATTTCGGTATGGCCCTCGCCCCATGGGATGTCATGGAGGTGGAAGATTTCAGAGTAA
ScAAD6-+1kb (1637) TGAACAGAGACTTCGAACGTGATATCATTCCAATGGCTAGGCATTTCGGTATGGCCCTCGCCCCATGGGATGTCATGGAGGTGGAAGATTTCAGAGTAA
      ScAAD6 (637) TGA-------------------------------------------------------------------------------------------------
     ScAAD14 (638) TGAACAGGGACTTTGAGCGTGATATTATTCCAATGGCTAGGCATTTTGGTATGGCTCTAGCCCCATGGGATGTCATGGAGGTGGAAGATTTCAGAGTAA
ScAAD14-+1lb (1638) TGAACAGGGACTTTGAGCGTGATATTATTCCAATGGCTAGGCATTTTGGTATGGCTCTAGCCCCATGGGATGTCATGGAGGTGGAAGATTTCAGAGTAA
      ScAAD4 (500) TGAACAGGGACTTTGAGCGCGATATCATTCCAATGGCCAGACATTTTGGTATGGCTCTAGCCCCATGGGATGTTATGGAGGTGGAAGATTTCAGAGTAA
ScAAD4-+1kb (1500) TGAACAGGGACTTTGAGCGCGATATCATTCCAATGGCCAGACATTTTGGTATGGCTCTAGCCCCATGGGATGTTATGGAGGTGGAAGATTTCAGAGTAA
```

```
      ScAAD10  (474) AAAGGCAGTGGAAGAGCGGAAGAAGAAAGGAGAAGGCTTGCGTACCTTTTTTGGTACTTCGGAACAGACGGATATGGAGGTTAAAATCAGCGAAGCATTG
ScAAD10-+1kb (1474) AAAGGCAGTGGAAGAGCGGAAGAAGAAAGGAGAAGGCTTGCGTACCTTTTTTGGTACTTCGGAACAGACGGATATGGAGGTTAAAATCAGCGAAGCATTG
      ScAAD15    (69) AAAAGCAATGGAGGAACGGAGGAAGAATGGAGAGTGTATTCGTTCTTTCGTTGGCGCCTCCGAACAAACAGATGCAGAAATCAAGATTAGTGAAGCATTA
ScAAD15-+1kb (1069) AAAAGCAATGGAGGAACGGAGGAAGAATGGAGAGTGTATTCGTTCTTTCGTTGGCGCCTCCGAACAAACAGATGCAGAAATCAAGATTAGTGAAGCATTA
 ScAAD3-+1kb (1729) AAAAGCAATGGAGGAACGGAGGAAGAATGGAGAGGGTATTCGTTCTTTCGTTGGCGCCTCCGAACAAACAGATGCAGAAATCAAGATTAGTGAAGCATTG
       ScAAD3   (729) AAAAGCAATGGAGGAACGGAGGAAGAATGGAGAGGGTATTCGTTCTTTCGTTGGCGCCTCCGAACAAACAGATGCAGAAATCAAGATTAGTGAAGCATTG
      ScAAD16    (69) AAAAGCAATGGAGGAACGGAGGAAGAATGGAGAGGGTATTCGTTCTTTCGTTGGCGCCTCTGAACAAACAGATGCAGAAATCAAGATTAGTGAAGCATTG
ScAAD16-+1kb (1069) AAAAGCAATGGAGGAACGGAGGAAGAATGGAGAGGGTATTCGTTCTTTCGTTGGCGCCTCTGAACAAACAGATGCAGAAATCAAGATTAGTGAAGCATTG
 ScAAD6-+1kb (1737) AAAAGCAATGGAGGAACGGAGGAAGAATGGAGAGGGTATTCGTTCTTTCGTTGGCGCCTCTGAACAAACAGATGCAGAAATCAAGATTAGTGAAGCATTG
       ScAAD6   (640) ----------------------------------------------------------------------------------------------------
      ScAAD14   (738) AAAAGCAATGGAAGAACGGAAGAAGAATGGAGAGGGTCTGCGTACTTTTGTGGGTGGCCCCGAACAAACAGAATTGGAGGTTAAAATCAGCGAAGCATTG
ScAAD14-+1lb (1738) AAAAGCAATGGAAGAACGGAAGAAGAATGGAGAGGGTCTGCGTACTTTTGTGGGTGGCCCCGAACAAACAGAATTGGAGGTTAAAATCAGCGAAGCATTG
       ScAAD4   (600) AAAAGCAATGGAGGAACGGAAGAAGAATGGAGAGGGTCTGCGTACTGTTTCGGGTACTTCTAAACAGACGGATAAAGAGGTTAAGATCAGTGAAGCATTG
 ScAAD4-+1kb (1600) AAAAGCAATGGAGGAACGGAAGAAGAATGGAGAGGGTCTGCGTACTGTTTCGGGTACTTCTAAACAGACGGATAAAGAGGTTAAGATCAGTGAAGCATTG

      ScAAD10   (574) TTAAAAGTTGCGGAAGAACATGGCACTGAGTCTGTCACTGCTATTGCCATAGCTTATGTTCGGTCTAAAGCGAAACATGTTTTCCCATTAGTGGGAGGAA
ScAAD10-+1kb (1574) TTAAAAGTTGCGGAAGAACATGGCACTGAGTCTGTCACTGCTATTGCCATAGCTTATGTTCGGTCTAAAGCGAAACATGTTTTCCCATTAGTGGGAGGAA
      ScAAD15   (169) GCCAAGGTTGCTGAGGAACATGGCACTGAGTCTGTTACTGCTATTGCTATTGCCTATGTTCGCTCTAAGGCGAAAAATGTTTTTCCGTCGGTTGAAGGAG
ScAAD15-+1kb (1169) GCCAAGGTTGCTGAGGAACATGGCACTGAGTCTGTTACTGCTATTGCTATTGCCTATGTTCGCTCTAAGGCGAAAAATGTTTTTCCGTCGGTTGAAGGAG
 ScAAD3-+1kb (1829) GCCAAGATTGCTGAGGAACATGGCACTGAGTCTGTTACTGCTATTGCTATTGCCTATGTTCGCTCTAAGGCGAAAAATTTTTTTCCGTCGGTTGAAGGAG
       ScAAD3   (829) GCCAAGATTGCTGAGGAACATGGCACTGAGTCTGTTACTGCTATTGCTATTGCCTATGTTCGCTCTAAGGCGAAAAATTTTTTTCCGTCGGTTGAAGGAG
      ScAAD16   (169) GCCAAGGTTGCTGAGGAACATGGCACTGAATCTGTTACTGCTATTGCTATTGCCTATGTTCGCTCTAAGGCGAAAAATGTTTTTCCATTGGTTGGAGGAA
ScAAD16-+1kb (1169) GCCAAGGTTGCTGAGGAACATGGCACTGAATCTGTTACTGCTATTGCTATTGCCTATGTTCGCTCTAAGGCGAAAAATGTTTTTCCATTGGTTGGAGGAA
 ScAAD6-+1kb (1837) GCCAAGGTTGCTGAGGAACATGGCACTGAATCTGTTACTGCTATTGCTATTGCCTATGTTCGCTCTAAGGCGAAAAATGTTTTTCCATTGGTTGGAGGAA
       ScAAD6   (640) ----------------------------------------------------------------------------------------------------
      ScAAD14   (838) ACTAAAATTGCTGAGGAACATGGAACAGAGTCTGTTACTGCTATCGCTATTGCCTATGTTCGCTCTAAAGCGAAAAATGTTTTCCCATTGATTGGAGGAA
ScAAD14-+1lb (1838) ACTAAAATTGCTGAGGAACATGGAACAGAGTCTGTTACTGCTATCGCTATTGCCTATGTTCGCTCTAAAGCGAAAAATGTTTTCCCATTGATTGGAGGAA
       ScAAD4   (700) GCCAAGGTTGCTGAGGAACATGGCACTGAGTCTGTTACTGCTATTGCTATTGCCTATGTTCGCTCTAAGGCGAAAAATGTTTTCCCATTGGTTGGTGGAA
 ScAAD4-+1kb (1700) GCCAAGGTTGCTGAGGAACATGGCACTGAGTCTGTTACTGCTATTGCTATTGCCTATGTTCGCTCTAAGGCGAAAAATGTTTTCCCATTGGTTGGTGGAA

      ScAAD10   (674) GAAAGATCGAACATCTCAAACAGAACATTGAGGCTTTGAGCATTAAATTAACACCAGAACAAATAAAGTACTTAGAAAGTATTGTTCCTTTTGATGTCGG
ScAAD10-+1kb (1674) GAAAGATCGAACATCTCAAACAGAACATTGAGGCTTTGAGCATTAAATTAACACCAGAACAAATAAAGTACTTAGAAAGTATTGTTCCTTTTGATGTCGG
      ScAAD15   (269) GAAAAATTGAGGATCTCAAAGAGAACATTAAGGCTCTCAGTATCGATCTAACGCCGGACAATATAAAATACTTGGAAAATGTAGTTCCTTTTGACATCGG
ScAAD15-+1kb (1269) GAAAAATTGAGGATCTCAAAGAGAACATTAAGGCTCTCAGTATCGATCTAACGCCGGACAATATAAAATACTTGGAAAATGTAGTTCCTTTTGACATCGG
 ScAAD3-+1kb (1929) GAAAAATTGAGGATCTCAAAGAGAACATTAAGGCTCTCAGTATCGATCTAACGCCAGACAATATAAAATACTTAGAAAGTATAGTTCCTTTTGACATCGG
       ScAAD3   (929) GAAAAATTGAGGATCTCAAAGAGAACATTAAGGCTCTCAGTATCGATCTAACGCCAGACAATATAAAATACTTAGAAAGTATAGTTCCTTTTGACATCGG
      ScAAD16   (269) GGAAAATTGAACACCTCAAACAGAACATTGAGGCTTTGAGCATTAAATTAACACCAGAACAAATAAAGTACTTAGAAAGTATTATTCCTTTTGATGTTGG
ScAAD16-+1kb (1269) GGAAAATTGAACACCTCAAACAGAACATTGAGGCTTTGAGCATTAAATTAACACCAGAACAAATAAAGTACTTAGAAAGTATTATTCCTTTTGATGTTGG
 ScAAD6-+1kb (1937) GGAAAATTGAACACCTCAAACAGAACATTGAGGCTTTGAGCATTAAATTAACACCAGAACAAATAAAGTACTTAGAAAGTATTATTCCTTTTGATGTTGG
       ScAAD6   (640) ----------------------------------------------------------------------------------------------------
      ScAAD14   (938) GGAAAATTGAACATCTCAAGCAGAACATTGAGGCTTTGAGTATTAAATTAACACCGGAACAAATAGAATACCTGGAAAGTATTGTTCCTTTTGATGTTGG
```

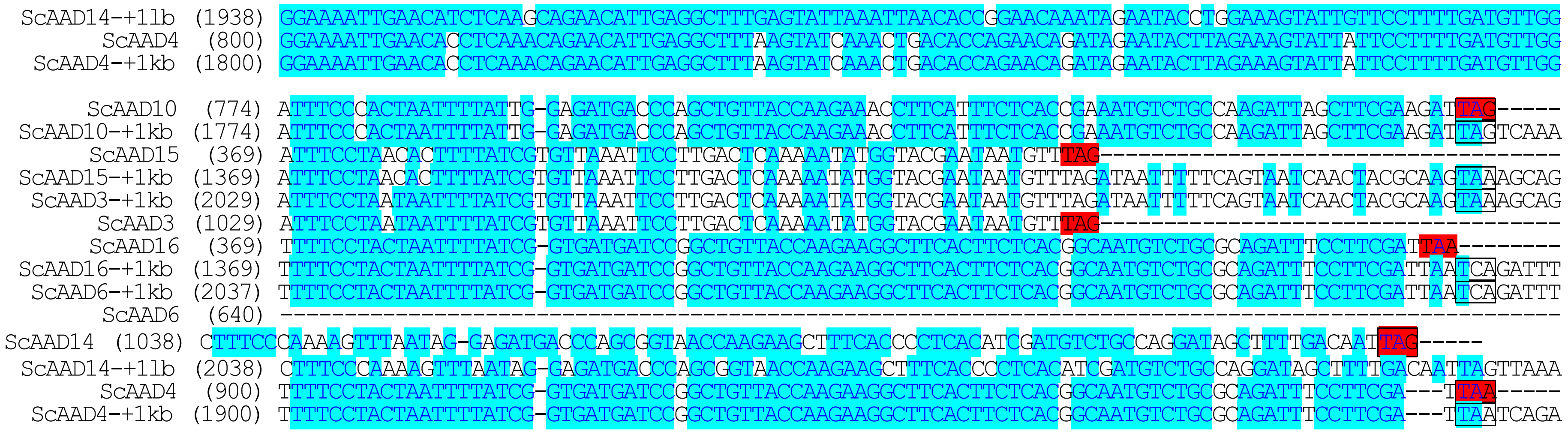

FIG S1 (A). Nucleotide sequence alignment of the putative *AAD* ORFs in *S. cerevisiae* lab strain S288C. Alignment shows that several out-of-frame sequences share still high homology with other *AADs*. SGD-defined start codons are shown in green and stop codons are in red. Hypothetical 'ancestral' start and stop codons are shown in black boxes. Consensus sequences are highlighted in blue.

FIG S1 (B)

```
   PcAad1p    (1) -MNIWAPAPEPPTKLGRHRQLAPGCGLHVSPIQLGAMSIGDKWHPYGMGTMDKEASFKLLDAFYNAGGNFIDTANVYQDE
  ScAad15p    (1) --------------------------------------------------------------------------------
   ScAad6p    (1) MADLFAPAPEPSTELGRLRVLSKSAGIRVSPLILGGMSIGDAWSE-ILGSMSKERAFELLDAFYEAGGNFIDTANNYQNE
  ScAad16p    (1) --------------------------------------------------------------------------------
   ScAad3p    (1) ---MIGSASDSSSKLGRLRFLSETAAIKVSPLILGEVSYDGARSD-FLKSMNKNRAFELLDTFYEAGGNFIDAANNCQNE
  ScAad10p    (1) --------------------------------------------------------------------------------
  ScAad14p    (1) MTDLFKPLPEPPTELGRLRVLSKTAGIRVSPLILGGASIGDAWSG-FMGSMNKEQAFELLDAFYEAGGNCIDTANSYQNE
   ScAad4p    (1) -----------------------------------------------MGSMNKEQAFELLDAFYEAGGNCIDTANSYQNE

   PcAad1p   (80) TSEEFIGEWMEARGNRDQMVVATKYSLVYKRGASFEEIPQKTQYVGNSLKSMHISVHDSLRKLRTSYIDIFYVHFWDYTC
  ScAad15p    (1) --------------------------------------------------------------------------------
   ScAad6p   (80) QSEAWIGEWMVSRKLRDQIVIATKFTTDYKKYDVGG--GKSANYCGNHKRSLHVSVRDSLRKLQTDWIDILYVHWWDYMS
  ScAad16p    (1) --------------------------------------------------------------------------------
   ScAad3p   (77) QSEEWIGEWIQSRRLRDQIVIATKFIKSDKKYKAGE--SNTANYCGNHKRSLHVSVRDSLRKLQTDWIDILYVHWWDYMS
  ScAad10p    (1) ---------MASRKLRDQIVIATKFTTDYKGYDVGK--GKSANFCGNHKRSLHVSVRDSLRKLQTDWIDILYVHWWDYMS
  ScAad14p   (80) ESEIWIGEWMASRKLRDQIVIATKFTGDYKKYEVGG--GKSANYCGNHKRSLHVSVRDSLRKLQTDWIDILYIHWWDYMS
   ScAad4p   (34) ESEIWIGEWMKSRKLRDQIVIATKFTGDYKKYEVGG--GKSANYCGNHKHSLHVSVRDSLRKLQTDWIDILYVHWWDYMS

   PcAad1p  (160) TIEEVMNGLHNLVAQGKVLYLGVSDTPAWVVSKANNYARMAGKTPFVIYEGEWNITMRDMERDIIPMCIHEGMAIAPWNV
  ScAad15p    (1) ------------------------------------------------------------------MARHFGMALAPWDV
   ScAad6p  (158) SIEEVMDSLHILVQQARSSIWVCLIRLPGLFLRQITTLNLMVKPLLASIKVNGTC-------------------------
  ScAad16p    (1) ------------------------------------------------------------------MARHFGMALAPWDV
   ScAad3p  (155) SIEEFMDSLHILVQQGKVLYLGVSDTPAWVVSAANYYATSYGKTPFSIYQGKWNVLNRDFERDIIPMARHFGMALAPWDV
  ScAad10p   (70) SIEEVMDSLHILVQQGKVLYLGVSDTPAWVVSAANYYATSHGKTPFSIYQGKWNVLNRDFERDIIPMARHFGMALAPWDV
  ScAad14p  (158) SIEEVMDSLHILVQQGKVLYLGVSDTPAWVVSAANYYATSHGKTPFSVYQGKWNVLNRDFERDIIPMARHFGMALAPWDV
   ScAad4p  (112) SIEEVMDSLHILVQQGKVLYLGVSDTPAWVVSAANYYATSHGKTPFSIYQGKWNVLNRDFERDIIPMARHFGMALAPWDV
 Consensus  (161) SIEEVMDSLHILVQQGKVLYLGVSDTPAWVVSAANYYATSHGKTPFSIYQGKWNVLNRDFERDIIPMARHFGMALAPWDV

   PcAad1p  (240) LCAGKIRTDAEEERRLKSGEGGRTLLQFDGWLRNETERKVSKALEKVAEEIGAKSITSVAIAYLMQKFPYVFPIVGGRKV
  ScAad15p   (15) MGGGRFQSKKAMEERRKNGECIRSFVG--ASEQTDAEIKISEALAKVAEEHGTESVTAIAIAYVRSKAKNVFPSVEGGKI
   ScAad6p  (213) --------------------------------------------------------------------------------
  ScAad16p   (15) MGGGRFQSKKAMEERRKNGEGIRSFVG--ASEQTDAEIKISEALAKVAEEHGTESVTAIAIAYVRSKAKNVFPLVGGRKI
   ScAad3p  (235) MGGGRFQSKKAMEERRKNGEGIRSFVG--ASEQTDAEIKISEALAKIAEEHGTESVTAIAIAYVRSKAKNFFPSVEGGKI
  ScAad10p  (150) MGGGRFQSKKAVEERKKKGEGLRTFFG--TSEQTDMEVKISEALLKVAEEHGTESVTAIAIAYVRSKAKHVFPLVGGRKI
  ScAad14p  (238) MGGGRFQSKKAMEERKKNGEGLRTFVG--GPEQTELEVKISEALTKIAEEHGTESVTAIAIAYVRSKAKNVFPLIGGRKI
   ScAad4p  (192) MGGGRFQSKKAMEERKKNGEGLRTVSG--TSKQTDKEVKISEALAKVAEEHGTESVTAIAIAYVRSKAKNVFPLVGGRKI
 Consensus  (241) MGGGRFQSKKAMEERKKNGEGIRTFVG  ASEQTD EIKISEALAKVAEEHGTESVTAIAIAYVRSKAKNVFPLVGGRKI

   PcAad1p  (320) EHLYANLEALDISLSPEQMQFLNDTVPFNKGFPYLLFGDGSDYNIVHKAAGHYDKWPAQQAIRPQK
```

```
ScAad15p   (93) EDLKENIKALSIDLTPDNIKYLENVVPFDIGFPNTFIVLNS--LTQKYGTNNV-------------
 ScAad6p  (213) ------------------------------------------------------------------
ScAad16p   (93) EHLKQNIEALSIKLTPEQIKYLESIIPFDVGFPTNFIGDDP--AVTKKASL-LTAMSAQISFD---
 ScAad3p  (313) EDLKENIKALSIDLTPDNIKYLESIVPFDIGFPNNFIVLNS--LTQKYGTNNV-------------
ScAad10p  (228) EHLKQNIEALSIKLTPEQIKYLESIVPFDVGFPTNFIGDDP--AVTKKPSF-LTEMSAKISFED--
ScAad14p  (316) EHLKQNIEALSIKLTPEQIEYLESIVPFDVGFPKSLIGDDP--AVTKKLSP-LTSMSARIAFDN--
 ScAad4p  (270) EHLKQNIEALSIKLTPEQIEYLESIIPFDVGFPTNFIGDDP--AVTKKASL-LTAMSAQISFD---
```

FIG S1 (B). Amino acid sequence alignment between *Pc*Aad1p (*Phanerochaete chrysosporium* Aad1p from strain BKM-F-1767) and putative *Sc*Aadp of S288C, as assigned in the current SGD. Identical residues are highlighted in blue; consensus sequences are highlighted in green.

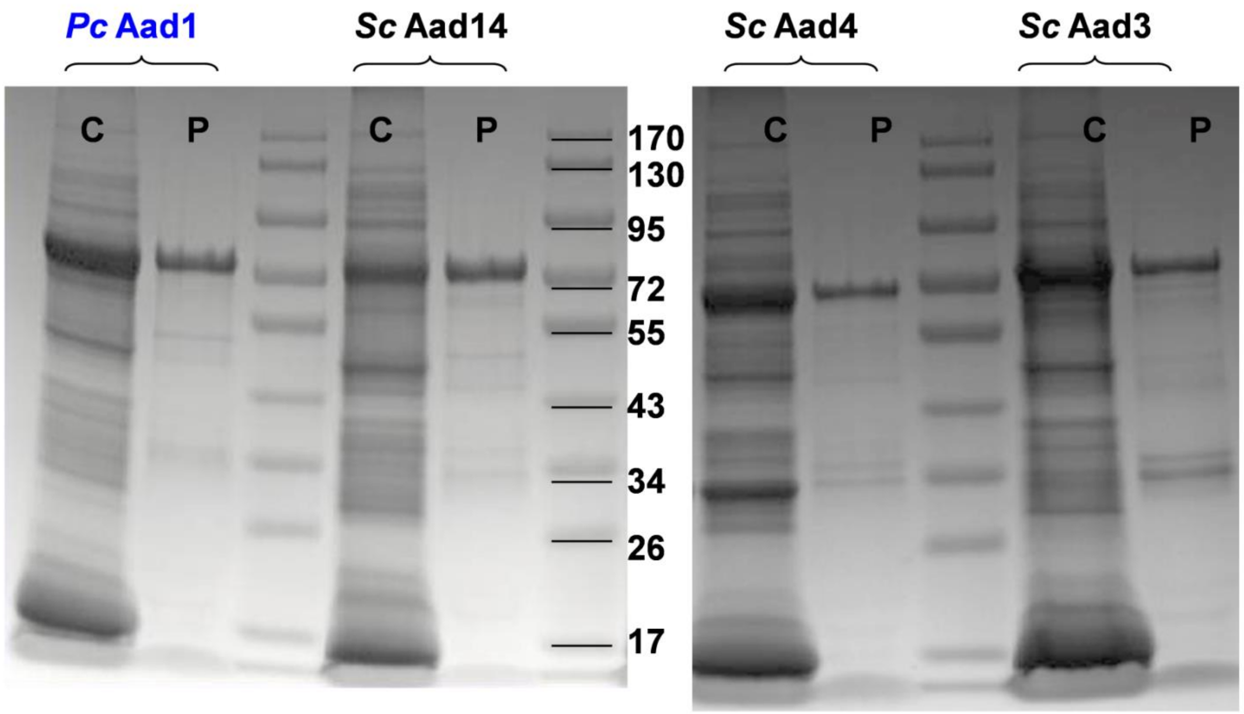

FIG S2. Heterologous expression and GST-affinity batch purifications of *Sc*Aad3p, *Sc*Aad4p, *Sc*Aad14p, and *Pc*Aad1p. "C" denotes crude extract, "P" indicates for the first protein elution following purification. Proteins were separated by SDS-PAGE, and stained with Coomassie Brilliant Blue G-250.

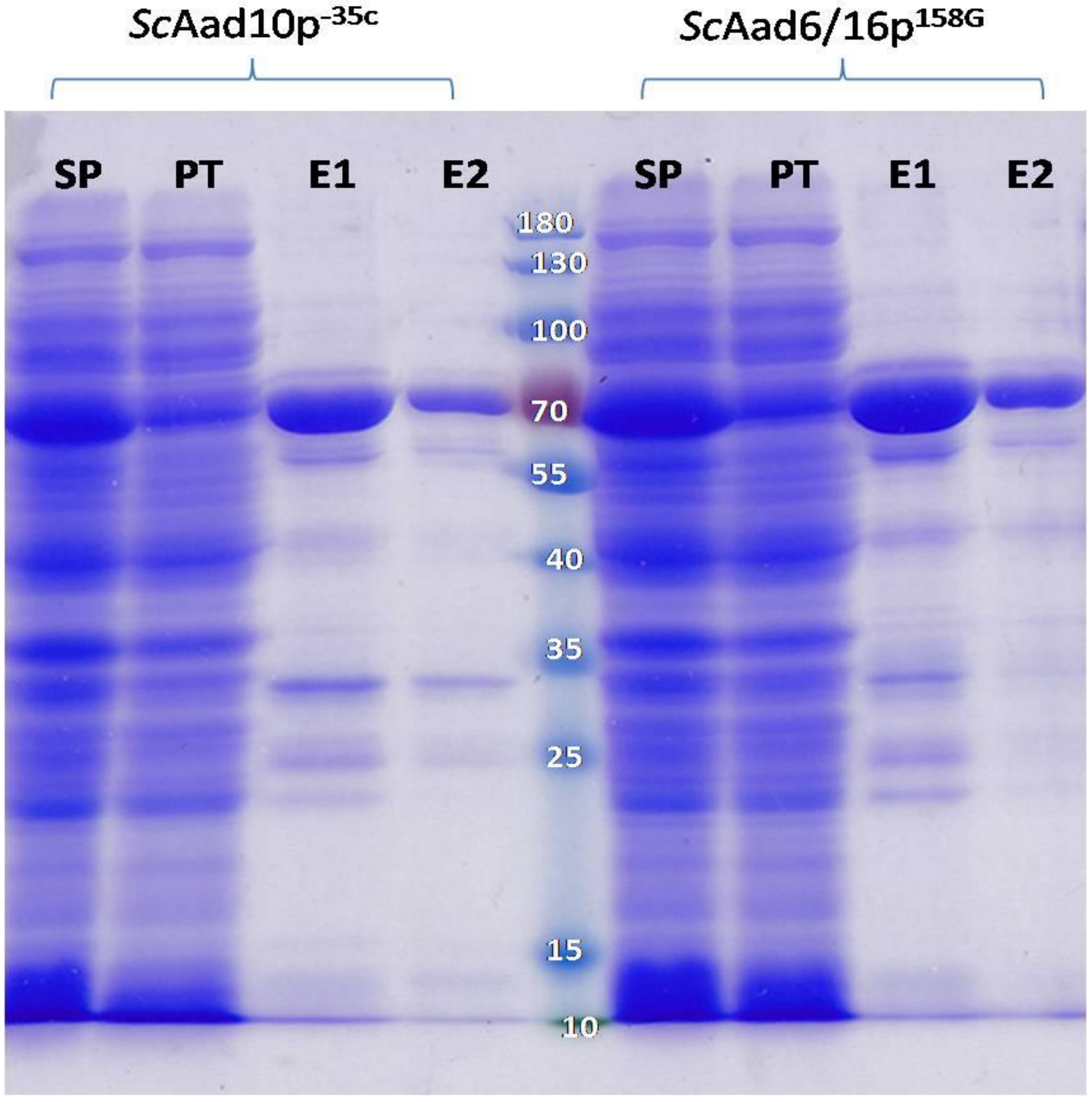

FIG S3. Heterologous expression and GST-affinity batch purification of reconstructed *Sc*Aad10^{-35C}p and *Sc*Aad6/16^{158G}p. **"SP"** indicates supernatant of crude extract, "**PT**" indicates the 1st bed volume (2 mL) pass-through elution, "E1" stands for 1st bed volume elution containing 10 mM reduced glutathione, "E2" stands for 2nd bed volume elution. Proteins were separated by SDS-PAGE and stained with Coomassie Brilliant Blue G-250.

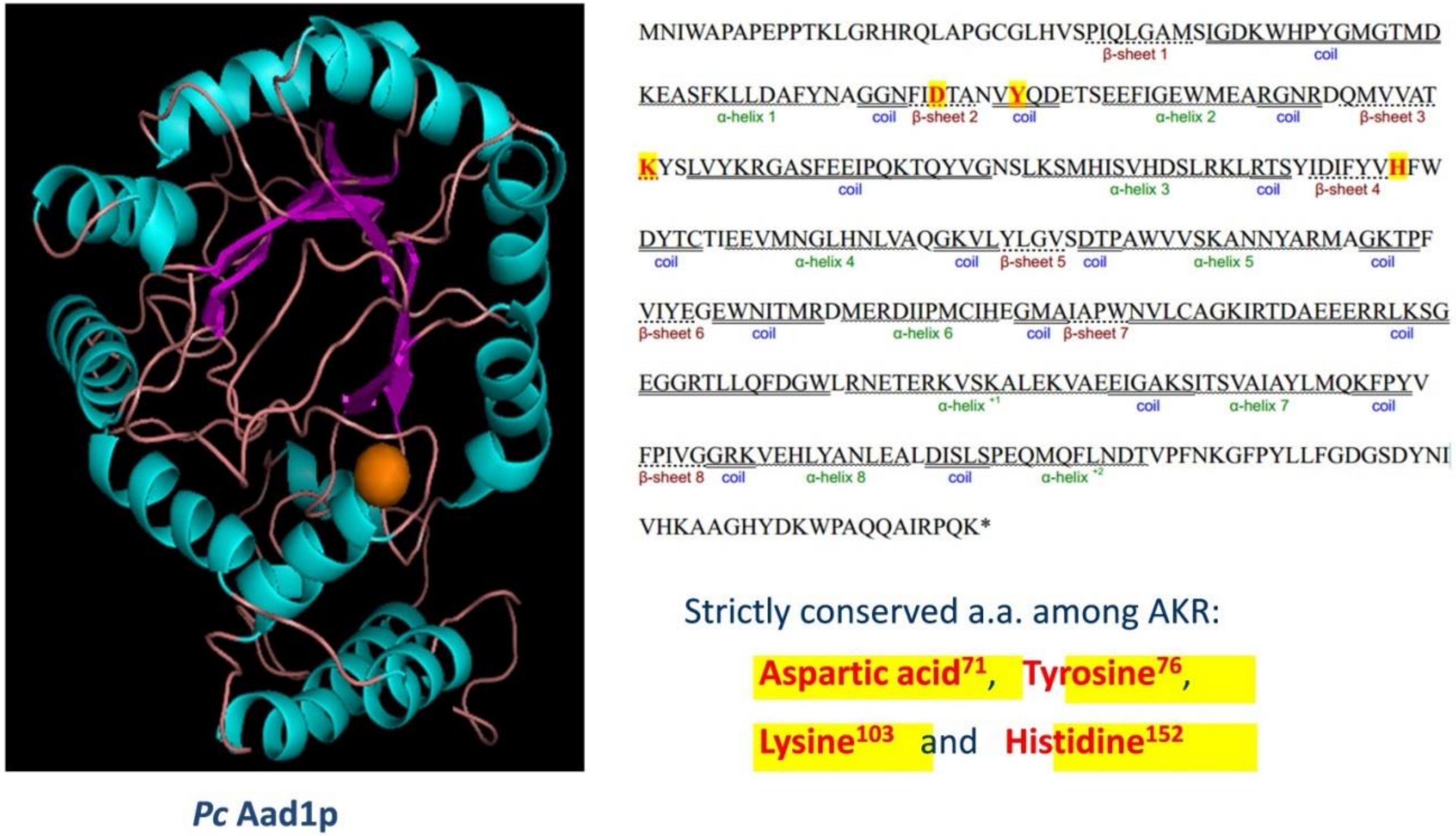

FIG S4 Structural analysis of the reference *Pc*Aad1p. *In silico* modeling reveals a (αβ)8-barrel motif typical of aldo-keto reductases. The α-helix is indicated in blue, β-sheet in purple. Two additional overhanging α-helices (at bottom of Fig. S4) and a sodium atom (in orange) are also noted. Within the catalytic tetrad, tyrosine acts as an acid in the reduction reaction, while the two additional amino acids complete a catalytic triad that activates the phenolic proton on tyrosine (Penning, TM, The aldo-keto reductase (AKRs): Overview. Chem Biol Interact. 234: 236-246, 2015; DOI : 10.1016/j.cbi.2014.09.024)

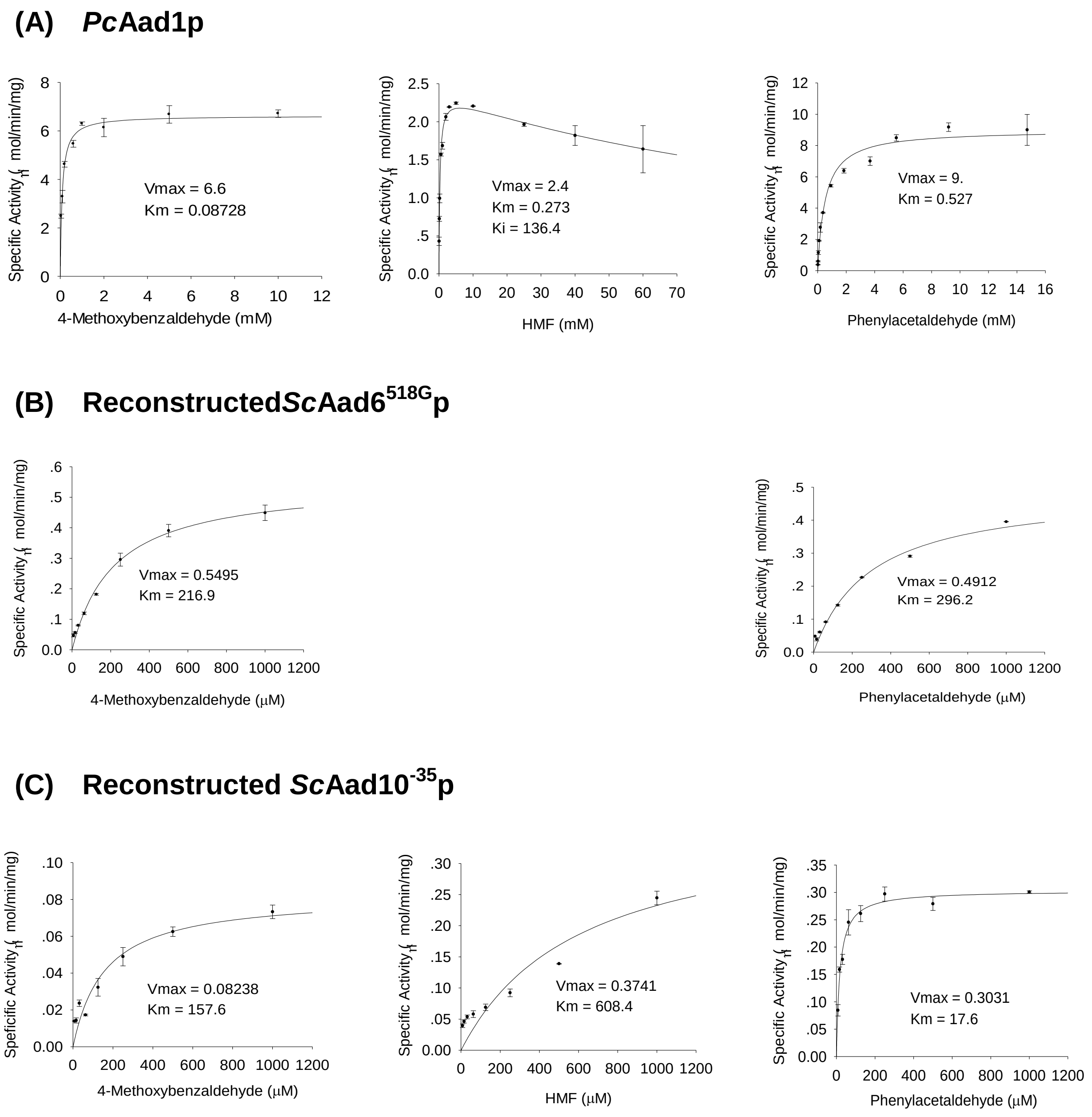

FIG S5. Recovery of enzyme activity from pseudogenized *Sc*Aad10 and *Sc*Aad6/16 following ancestral state reconstruction. Kinetic data were obtained by assaying purified enzymes in MES buffer (50 mM, pH 6.1) containing 0.3 mM NADPH. Each data point represents the mean $\pm$ SEM of triplicate experiments.

FIG S6 (A)

ScAAD6^{518G}	(1)	ATGGCTGATTTATTTGCTCCTGCTCCTGAACCATCTACAGAGTTAGGACGTCTCAGAGTTCTTTCTAAAAGTGCTGGTAT
T7 AAD6	(1)	ATGGCTGATTTATTTGCTCCTGCACCTGAACCACCTACCGAGTTAGGACGTCTTAGGGTCCTATCTAAAACAGCTGGTAT
ScAAD6^{518G}	(81)	TAGAGTTTCCCCTCTCATTTTGGGAGGAATGTCAATTGGCGACGCTTGGTCTGAAATCCTAGGATCAATGAGCAAGGAGC
T7 AAD6	(81)	AAGAGTTTCTCCACTAATCCTGGGAGGTATGTCTATTGGTGACGCCTGGTCTGGATTCATGGGATCAATGGACAAAGAAC
ScAAD6^{518G}	(161)	GAGCTTTTGAGTTGCTCGATGCTTTTTATGAGGCAGGTGGAAATTTCATTGATACTGCAAATAATTATCAAAACGAACAG
T7 AAD6	(161)	AAGCTTTTGAGCTACTTGATGCTTTTTACCAAGCAGGCGGAAATTTCATTGATACTGCAAATAATTATCAAAACGAACAG
ScAAD6^{518G}	(241)	TCAGAAGCTTGGATCGGTGAATGGATGGTTTCAAGAAAATTACGCGACCAGATTGTAATCGCCACCAAGTTTACCACTGA
T7 AAD6	(241)	TCAGAAGCTTGGATCGGTGAATGGATGGTTTCAAGAAAATTACGCGACCAGATTGTAATCGCCACCAAGTTTACCATTGA
ScAAD6^{518G}	(321)	CTATAAGAAATACGACGTTGGTGGTGGTAAAAGCGCAAACTACTGTGGCAATCACAAGCGTAGTTTGCATGTGAGTGTGA
T7 AAD6	(321)	CTATAAGAAATACGACGTTGGTGGTGGTAAAAGCGCAAACTACTGTGGCAATCACAAGCGTAGTTTGCATGTGAGTGTGA
ScAAD6^{518G}	(401)	GGGATTCTCTCCGCAAATTGCAAACTGATTGGATTGATATACTTTACGTTCACTGGTGGGATTATATGAGTTCTATCGAA
T7 AAD6	(401)	GGGATTCTCTCCGCAAATTGCAAACTGATTGGATTGATATACTTTACGTTCACTGGTGGGATTATATGAGTTCTATCGAA
ScAAD6^{518G}	(481)	GAAGTTATGGATAGTTTACACATTCTAGTGCAGCAGGGCAAGGTCCTCTATTTGGGTGTGTCTGATACGCCTGCCTGGGT
T7 AAD6	(481)	GAAGTTATGGATAGTTTACACATTCTTGTGCAGCAGGGCAAAGTCCTCTATCTAGGTGTGTCTGATACGCCTGCCTGGGT
ScAAD6^{518G}	(561)	TGTTTCTGCGGCAAATTACTACGCTAAATCTCATGGTAAAACCCCTTTTAGCATCTATCAAGGTAAATGGAACCTGTTGA
T7 AAD6	(561)	TGTTTCTGCGGCAAATTACTACACTAAATCTCATGGTAAAACCCCTTTTAGCATCTATCAAGGTAAATGGAACCTGTTGA
ScAAD6^{518G}	(641)	ACAGAGACTTCGAACGTGATATCATTCCAATGGCTAGGCATTTCGGTATGGCCCTCGCCCCATGGGATGTCATGGGAGGT
T7 AAD6	(641)	ACAGAGACTTCGAACGTGATATCATTCCAATGGCTAGGCATTTCGGTATGGCCCTCGCCCCATGGGATGTCATGGGAGGT
ScAAD6518GG	(721)	GGAAGATTTCAGAGTAAAAAAGCAATGGAGGAACGGAGGAAGAATGGAGAGGGTATTCGTTCTTTCGTTGGCGCCTCTGA
T7 AAD6	(721)	GGAAGATTTCAGAGTAAAAAAGCAATGGAGGAACGGAGGAAGAATGGAGAGGGTATTCGTTCTTTCGTTGGCGCCTCTGA
ScAAD6^{518G}	(801)	ACAAACAGATGCAGAAATCAAGATTAGTGAAGCATTGGCCAAGGTTGCTGAGGAACATGGCACTGAATCTGTTACTGCTA
T7 AAD6	(801)	ACAAACAGATGCAGAAATCAAGATTAGTGAAGCATTGGCCAAGGTTGCTGAGGAACATGGCACTGAATCTGTTACTGCTA
ScAAD6^{518G}	(881)	TTGCTATTGCCTATGTTCGCTCTAAGGCGAAAAATGTTTTTCCATTGGTTGGAGGAAGGAAAATTGAACACCTCAAACAG
T7 AAD6	(881)	TTGCTATTGCCTATGTTCGCTCTAAGGCGAAAAATGTTTTCCCATTGGTTGGAGGAAGGAAAATTGAACACCTCAAACAG

```
ScAAD6518G (961)  AACATTGAGGCTTTGAGCATTAAATTAACACCAGAACAAATAAAGTACTTAGAAAGTATTATTCCTTTTGATGTTGGTTT
   T7 AAD6 (961)  AACATTGAGGCTTTAAGTATCAAACTGACACCAGAACAGATAGAATACTTAGAAAGTATTATTCCTTTTGATGTTGGTTT

ScAAD6518G (1041) TCCTACTAATTTTATCGGTGATGATCCGGCTGTTACCAAGAAGGCTTCACTTCTCACGGCAATGTCTGCGCAGATTTCCT
   T7 AAD6 (1041) TCCTGCTAATTTTATCGGTGATGATCCGGCTGTTACCAAGAAGGCTTCATTTCTCACGGCAATGTCTGCGCAGATTTCCT

ScAAD6518G (1121) TCGATTAA
   T7 AAD6 (1121) TCGATTAA
```

FIG S6 (B)

```
ScAad6p518G (1)   MADLFAPAPEPSTELGRLRVLSKSAGIRVSPLILGGMSIGDAWSEILGSMSKERAFELLDAFYEAGGNFIDTANNYQNEQ
   T7 Aad6p (1)   MADLFAPAPEPPTELGRLRVLSKTAGIRVSPLILGGMSIGDAWSGFMGSMDKEQAFELLDAFYQAGGNFIDTANNYQNEQ

ScAad6p518G (81)  SEAWIGEWMVSRKLRDQIVIATKFTTDYKKYDVGGGKSANYCGNHKRSLHVSVRDSLRKLQTDWIDILYVHWWDYMSSIE
   T7 Aad6p (81)  SEAWIGEWMVSRKLRDQIVIATKFTIDYKKYDVGGGKSANYCGNHKRSLHVSVRDSLRKLQTDWIDILYVHWWDYMSSIE

ScAad6p518G (161) EVMDSLHILVQQGKVLYLGVSDTPAWVVSAANYYAKSHGKTPFSIYQGKWNLLNRDFERDIIPMARHFGMALAPWDVMGG
   T7 Aad6p (161) EVMDSLHILVQQGKVLYLGVSDTPAWVVSAANYYTKSHGKTPFSIYQGKWNLLNRDFERDIIPMARHFGMALAPWDVMGG

ScAad6p518G (241) GRFQSKKAMEERRKNGEGIRSFVGASEQTDAEIKISEALAKVAEEHGTESVTAIAIAYVRSKAKNVFPLVGGRKIEHLKQ
   T7 Aad6p (241) GRFQSKKAMEERRKNGEGIRSFVGASEQTDAEIKISEALAKVAEEHGTESVTAIAIAYVRSKAKNVFPLVGGRKIEHLKQ

ScAad6p518G (321) NIEALSIKLTPEQIKYLESIIPFDVGFPTNFIGDDPAVTKKASLLTAMSAQISFD-
   T7 Aad6p (321) NIEALSIKLTPEQIEYLESIIPFDVGFPANFIGDDPAVTKKASFLTAMSAQISFD-
```

FIG S6. A full-length *AAD6* identified in *S. cerevisiae* T7 strain living in oak tree exudate. This *AAD6* exhibits >95% consensus positions with the reconstructed hypothetical *AAD6*. **(A)** and **(B)** show the alignment of nucleic acids and the amino acid sequences they encode.

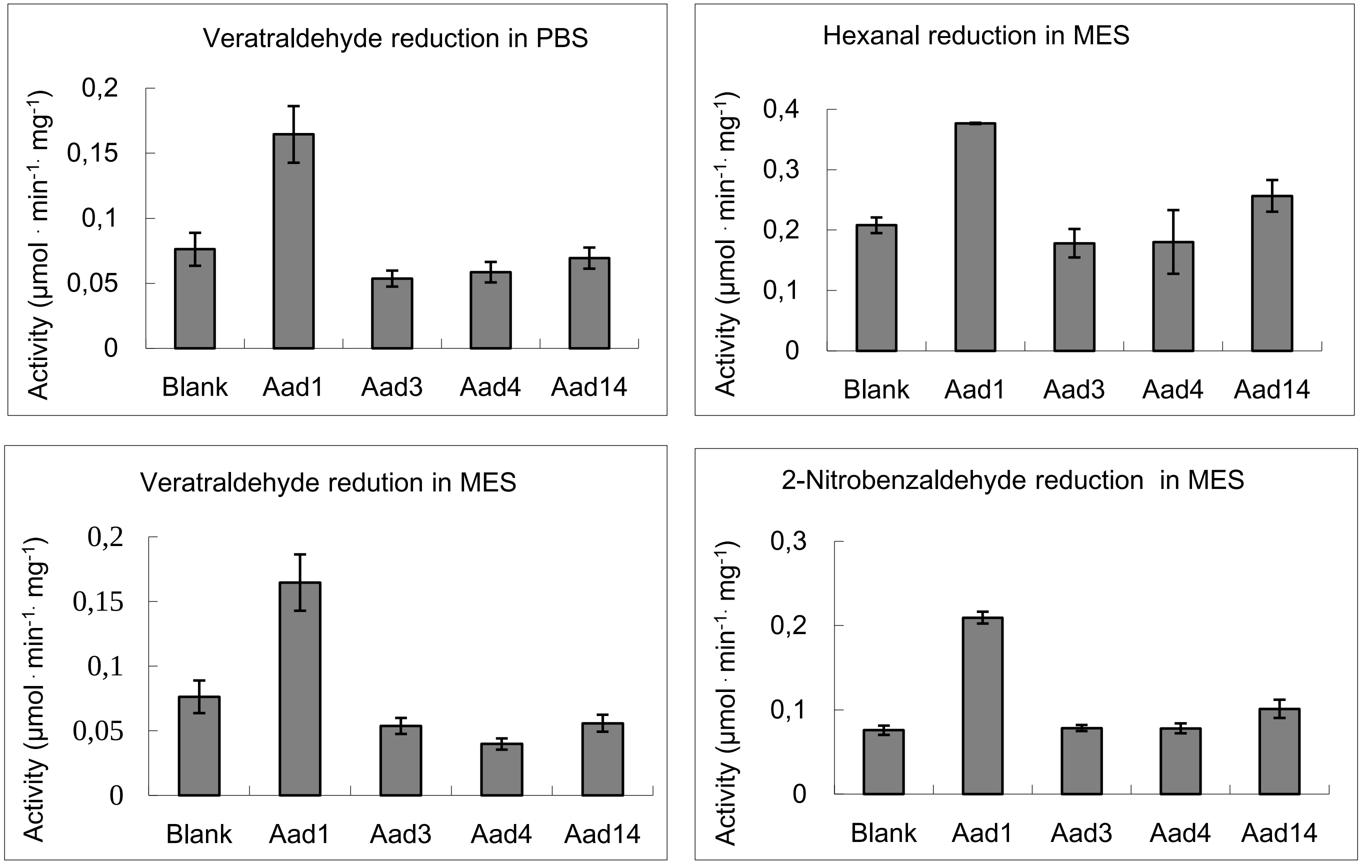

FIG S7. Aldehyde reduction activity measured with *S. cerevisiae* crude enzyme extract overexpressing Aad. BY4741 strains harbors “blank” or Aad (“Aad1”, “Aad3”, “Aad4”, “Aad14”) YEPlac 195 vectors. Activity was assayed on crude cell extracts in 250 µL MES buffer (50 mM, pH 6.1) containing 0.3 mM NADPH, 0.3 mM substrates and 20 µl crude extract. Veratraldehyde (3,4-dimethoxybenzaldehyde), hexanal and 2-Nitrobenzaldehyde were used as they are the most reactive substrates (Fig 1) for purified *Pc*Aad1p, *Sc*Aad4p and *Sc*Aad14p.

TABLE S1. High variability of *AAD* ORFs in sequenced *S. cerevisiae* genomes, as compared to other non-telomeric aldehyde reductases.

Strains / Query	**BY4741**	**YJM789**	**EC1118**	**AWRI1631**	**AWRI796**	**Lalvin QA23**	**VL3**	**FostersB**	**FostersO**	**Kyokai no. 7**	**Sigma1278b**
	Lab	Lab	Wine	Wine	Wine	Wine	Wine	Beer	Beer	Sake	Pathologic
AAD3	100/100	96.4/93.7	99.8/99.5	99.9/99.5	≥99.4/82.1	99.7/67.6	99.8/85.2	N.R.	N.R.	N.R.	98.2/82.7
AAD4^{-136G}	99.9/83.5	93.2/93.9	94.3/84.6	94.4/84.3	94.3/84.3	94.2/71.3	94.2/84.3	95.9/84.3	94.3/84.3	93.4/94.7	N.R.
AAD6^{518G}	99.9/-	N.R.	N.R.	N.R.	N.R.	N.R.	N.R.	N.R.	N.R.	99.8/-	N.R.
AAD10^{-35C}	99.9/76.4	99.9/76.4	99.7/76.4	99.8/76.1	99.6/99.5	N.R.	92.6~93.2/72.9	N.R.	N.R.	*/*	87.3/55.2
AAD14	100/100	99.2/99.5	97.1/97.6	N.R.	97.1/97.6	96.9/52.0	96.9/65.3	97.3/97.1	97.5/97.3	100/100	100/100
AAD15	100/100	N.R.	97.7/95.1	N.R.	N.R.	N.R.	N.R.	N.R.	N.R.	N.R.	100/100
YPL088W	100/100	99.2/98.5	99.1/98.5	*/*	99.3/98.5	98.9/98.3	99.1/81.9	99.2/98.5	99.0/80.5	99.7/99.1	100/100
ADH1	100/100	98.6/98.6	99.0/98.6	98.6/98.6	98.6/98.3	98.9/98.6	98.6/98.6	98.7/98.6	98.4/98.0	98.9/98.6	100/100
ADH2	100/100	98.4/98.0	97.8/98.3	N.R.	97.8/98.3	97.7/43.0	97.8/98.3	97.2/97.7	94.1/95.4	99.5/99.1	N.R.
ADH3	100/100	100/100	100/100	100/100	100/100	100/100	100/100	99.7/99.5	99.7/99.5	99.9/100	100/100
ADH4	100/100	99.9/99.5	98.3/98.2	98.2/97.9	98.3/98.2	98.0/23.8	98.3/98.2	99.8/99.2	98.1/97.4	99.7/99.5	98.3/98.2
ADH5	100/100	99.6/100	99.6/100	99.4/99.4	99.6/100	99.6/100	99.6/100	99.5/99.4	99.7/99.4	99.9/100	100/100
ADH6	100/100	99.7/100	99.5/99.4	99.7/100	99.7/100	99.7/100	99.5/30.2	99.1/98.6	99.5/100	100/100	100/100
ADH7	100/100	99.8/99.4	100/100	100/100	100/100	100/100	100/100	N.R.	N.R.	N.R.	100/100
GRE3	100/100	100/100	99.9/99.4	100/100	100/10	99.9/99.4	100/100	99.9/99.4	99.8/87.8	99.7/100	100/100
GCY1	100/100	100/100	99.6/98.7	99.8/99.4	99.8/99.4	99.3/≤25.2	99.7/99.0	99.3/98.7	99.4/99.4	99.3/99.0	99.8/99.8
YPR1	100/100	99.3/12.8	100/100	100/100	99.8/93.6	100/100	99.6/99.4	99.7/99.4	99.6/67.4	99.8/99.4	100/100
YDL124W	100/100	99.4/98.4	99.4/98.4	99.0/97.4	99.0/97.4	99.4/98.4	99.3/98.4	99.8/99.0	99.4/98.7	98.0/70.0	98.4/97.1
YJR096W	100/100	99.4/99.3	100/100	99.9/48.4	100/100	99.9/86.2	100/100	99.9/100	99.9/100	99.6/98.9	100/100
ARA1	100/100	99.6/100	99.8/99.4	99.7/99.1	99.8/99.4	99.8/99.4	99.8/99.4	99.2/50.4	99.5/8.7	75.4/75.4	100/100

Nucleic acid/amino acid sequence percentage similarity relative to query sequences from lab strain S288C. N.R.: *AAD* homologs were not retrieved. Asterisks (*****): percentage similarity not calculated when only partial sequences were found at the end of a sequencing contig with missing 3' or 5' coding sequences.
